# Women, the heart of intuition, and creation entanglement

**DOI:** 10.3389/fpsyg.2025.1547229

**Published:** 2025-08-20

**Authors:** Raymond T. Bradley, Chaarenne T. Torris

**Affiliations:** Research Center, Institute for Whole Social Science, Northland, New Zealand

**Keywords:** women/mother/parental intuition, love, heart coherence, nonlocality/entanglement, quantum holography, electrophysiological measures, brain, consciousness

## Abstract

Within the broader topic of women's intuition, the focus of this work is mother-offspring intuition—the enigmatic connection between a mother and her offspring, wherein distress or imminent harm is instantly sensed, even when thousands of miles apart. Instant nonlocal communication irrespective of distance, is known as entanglement in quantum physics, and has been repeatedly verified since the 1970′s. Building upon this discovery, a new concept–*multiscale entanglement*–is postulated to create a two-way micro to macroscale bond of tacit connection between a mother and a fetus, initiated at conception. Nurtured by the mother's unconditional love and mediated by her self-induced state of heart coherence, the process evolves during gestation by entanglement among subatomic and atomic constituents of cells, extending to molecules, organs and so forth to create a tacit bond of ‘inseparability' between their bodies. By the time the offspring has reached adolescence, this relation has often become a two-way *psychic* bond whereby *either* can instantly intuit something is amiss or that imminent harm/danger awaits the other. A quantum holographic approach is used to describe the information communication processes involved, in which states of selfless/unconditional love create a psychoenergetic channel for nonlocal communication—of which mother-offspring intuition is one facet. The work is documented with evidence where available, and begins with a review of research on women's intuition. This includes an in-depth examination of a groundbreaking electrophysiological experiment on nonlocal intuition documenting brain and heart response to prestimulus information by gender.

If Goddess is something within everyone, and all life is part of her, there is oneness and unity of all the universe. Information is shared … to be tapped into by anyone open to it. *This tapping-in is a woman's work skill …. The skill is woman's intuition …*. Women's intuition is … a way of becoming part of the timeless and whereless space-time/Goddess continuum. … *Every act of psychic knowing is an act of women's intuition* ….

Stein (1988),

*All Women Are Psychics*, pages 15–16; italics added.

Love is a kind of exalted but unspecialized telepathy; the simplest and most universal expression of that mutual gravitation or kinship of spirits which is the foundation of the telepathic law.

Myers (1907),

*Human Personality and Its Survival of Bodily Death*, page 344.

Nonlocality [quantum entanglement] and the nonlocal quantum hologram provide the *only* testable mechanism discovered to date which offers a possible solution to the host of enigmatic observations and data associated with consciousness and such consciousness phenomena.

Mitchell (2000),

Nature's Mind: The Quantum Hologram, page 299; italics added.

Two particles that may be very far apart, even millions or billions of miles, are mysteriously linked together.... Entangled particles transcend space.... The [entangled] system acts as a single entity.... Spatial separation as we know it seems to evaporate …. What happens to one of them happens to the other one *instantaneously*, regardless of the distance between them.

Aczel (2001),

*Entanglement: The Greatest Mystery in Physics*,

pages xi, 252 & 250, respectively; italics added.

## Introduction

Intuition is a longtime focus of interest in psychology and social science and has been investigated for more than a century. Involving a broad spectrum of psychic abilities, evidence shows that intuition is a nonlocal proficiency possessed by *all* humans (Radin, 1997a).

These days intuition has become a “hot” topic of research. Most work is focused on the more commonly used notion of intuition—a cognition-based concept in which intuition is viewed as the brain's extrapolation from memories of prior experience (e.g., Agor, 1984; Hogarth, 2001; Myers, 2002). Since these studies rely on “self-report” responses to questionnaires or interviews, they measure *subjective* perceptions about intuition, rather than intuition itself (Lufityanto, 2020). There is, however, an alternative concept—*nonlocal intuition*, which is the body's perception of tacit information from remote objects or events that have yet to happen (McCraty et al., 2004a). This conception is based on laboratory-controlled experiments using *objective* measures of physiological response during the *pre*-stimulus period.

Despite a scarcity of studies on gender, there is evidence suggesting some notable differences between women and men. Namely, variances in intuitive (pre-stimulus) response, differences in the brain regions and locus of the psychophysiological systems involved. These appear to be linked to women's greater socioemotional sensitivity, which, while based on bio-genetic differences (Ramos-Loyo et al., 2022), are mediated by ethno-cultural factors (Eisler and Fry, 2019). Even so, in the absence of studies with larger gender samples, these results should be regarded as “working hypotheses”. Overall, the evidence shows that nonlocal proficiencies can be honed with experience and practice, and that psychic effects are strongest with whom we love or feel an attachment to. In this context, the psychic bond between mother and child appears a special but important case. In developing our account, we posit a union of *multiscale entanglement* between the subatomic and atomic constituents of the mother and fetus' bodies, which evolves to create a lifelong psychic bond by adolescence.

### Enigma of mother-offspring intuition

The *instant* intuitive bond between a mother and her offspring, even when they are thousands of miles apart (Stein, 1988), has remained a longstanding enigma. In her study of self-reported “psychic” experiences of 70 women, Diane Stein finds that:

Messages that matter, that come from someone loved, are the most likely to be received telepathically or sent. Most telepathy occurs between women who care about each other, have deep emotional ties, and know each other well. *The [psychic] link between mothers and children is a classic one* … (Stein, 1988, p. 178–179; italics added).

This raises a question of great importance: *how* is such a tacit bond of instant nonlocal communication established between a mother and her offspring?

Instant *nonlocal* communication irrespective of distance, is known as *entanglement* in quantum physics, and has been repeatedly verified in experiments since the 1970′s. It is a microscale phenomenon of superluminal communication observed among pairings of subatomic particles or atoms entangled in a nonlocal bond of “inseparability” (Aczel, 2001). In a recent work (Bradley, 2024c), the first author (RB) posits that a process of multiscale entanglement, initiated at conception, generates a nonlocal channel of connection between a mother and her offspring which is operative for life. In developing this approach, we suggest that multiscale entanglement is propelled by a mother's unconditional love. By adolescence, this tacit relation of inseparability is manifest as a two-way ever-present, always ready psychic connection that transcends spacetime and quantum reality. We illustrate these concepts from the second author's (CT) personal experience and two cases from her psychotherapy practice. Our goal is to illustrate the utility of this approach, both in furthering the scientific understanding of intuition and in highlighting its value for clinical practice. We begin with a highly condensed summary of the research on nonlocal intuition describing the psychophysiological systems involved, with an emphasis on gender differences.

## Nonlocal intuition

Nonlocal psychoenergetic communication is a well-documented phenomenon within and among all living systems (e.g., Sheldrake, 1999) and has been extensively investigated in humans for more than a century (Radin, 1997a). Evolving from precognition dice tossing studies and telepathic “forced choice” card transmission experiments (Rhine, 1964, 1981), today's researchers employ sophisticated computer-assisted experiments to investigate the full spectrum of human psychic proficiencies—including the sensory channels involved (Radin, 2006). The advent of computer technology also spawned a novel measurement methodology in which random number generators (RNGs) have been used in the Global Consciousness Project to register the impact of mass focus on world events on “global consciousness” (e.g., Nelson et al., 1998; Nelson, 2001; Bancel and Nelson, 2008) and to register the effects of activities/events on collective consciousness in groups (e.g., Mason et al., 2007; see Bradley, 2024e). Since then, sophisticated experiments adapting experiment protocols from quantum physics have shown that “focused intention” can change the behavior of subatomic particles (e.g., Radin et al., 2012, 2013).

### Psychophysiological basis

In an early effort to identify a physiological basis for nonlocal communication, (Tart 1963) found that the “receiver's” brain waves and peripheral blood volume changed when a stimulus was administered near to or on the “sender's” body. Schlitz and Braude (1997) conducted a meta-analysis of “staring” studies (popular at that time) and found significant Galvanic Skin Response (GSR) in the “receiver's” bodies when the “senders” thoughts were focused on them. Investigations of precognition examined the brain's prestimulus response and found significant differences in event-related potentials (ERPs) before target presentation (Warren et al., 1992a,b). Don and associates followed up in a series of studies using a gambling protocol (Don et al., 1998; McDonough et al., 2002), and because they found that enhanced negativity in the ERP's across the scalp appeared in response to future targets, they concluded that the “ERP effect” was an indicator of “unconscious precognition”.

In the past three decades, researchers have focused on *presentiment* to investigate the degree to which emotion mediated the body's response to and processing of tacit pre-stimulus information. Radin (1997a,b, 2004) designed elegant experiments to evoke an emotional response using randomly selected emotionally “arousing” or “calming” photographs, with measures of skin conductance level (SCL) and photoplethysmographic measures of heart rate and blood volume. He found significantly greater change in electrodermal activity around *5 seconds before* a future “emotional” picture was presented than before a “calm” one. These results have been replicated (e.g., Bierman and Radin, 1997; Bierman, 2000; Radin, 2004), and a follow-up *f* MRI study found brain activation near the amygdala (which modulates strong emotions such as fear and rage) *before* “emotional” pictures were shown, but not before “calm” ones (Bierman and Scholte, 2002).

### Brain and heart involvement

**Figure 1:**
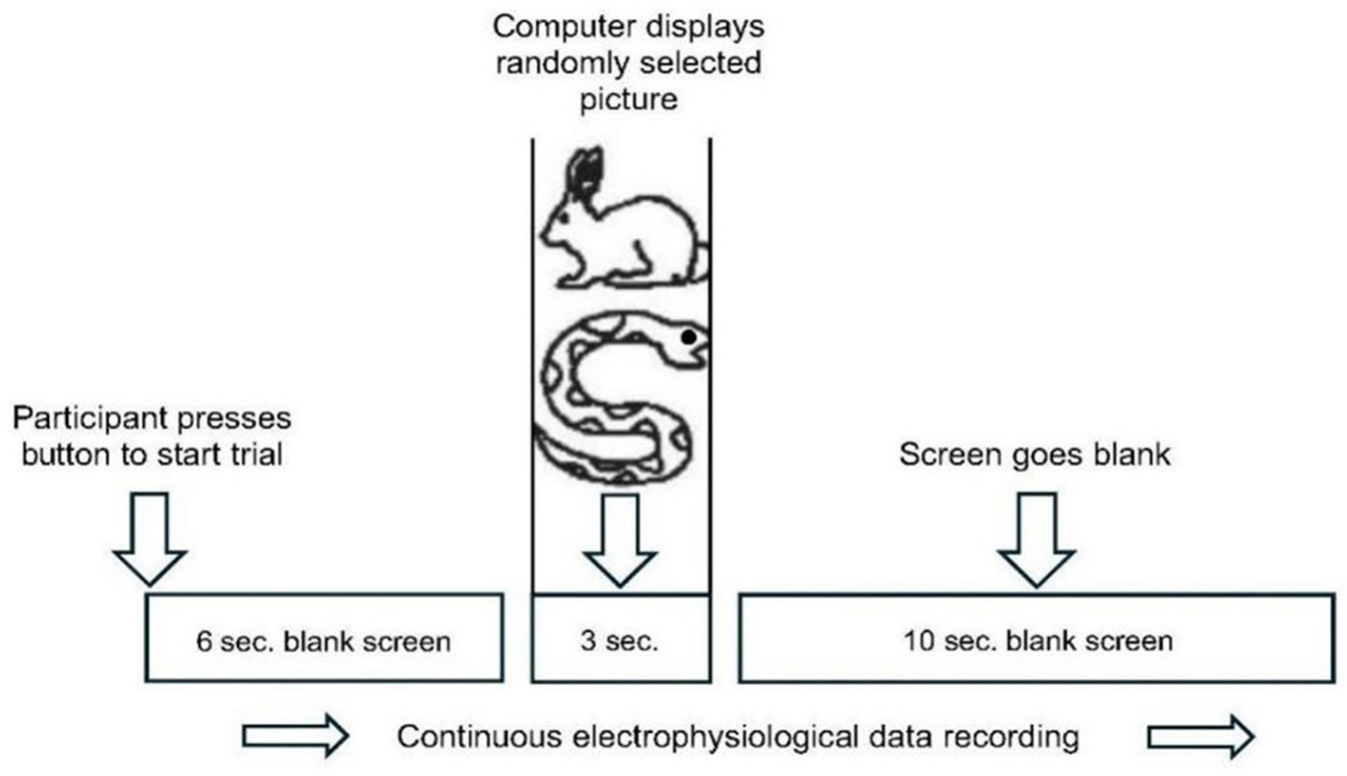
Experiment protocol. Seated comfortably in front of a computer screen, participant presses a button to begin each trial. The screen remains blank for 6 s. Then the computer presents a randomly selected image from either the calm (e.g., rabbit) or emotional (e.g., snake) picture sets, displaying it for 3 s. A blank screen follows for 10 s. After this resting period, a screen message instructs participants to begin the next trial, when ready (adapted from McCraty et al., Fig. 1, 2004a ©; used with permission).

**Figure 2:**
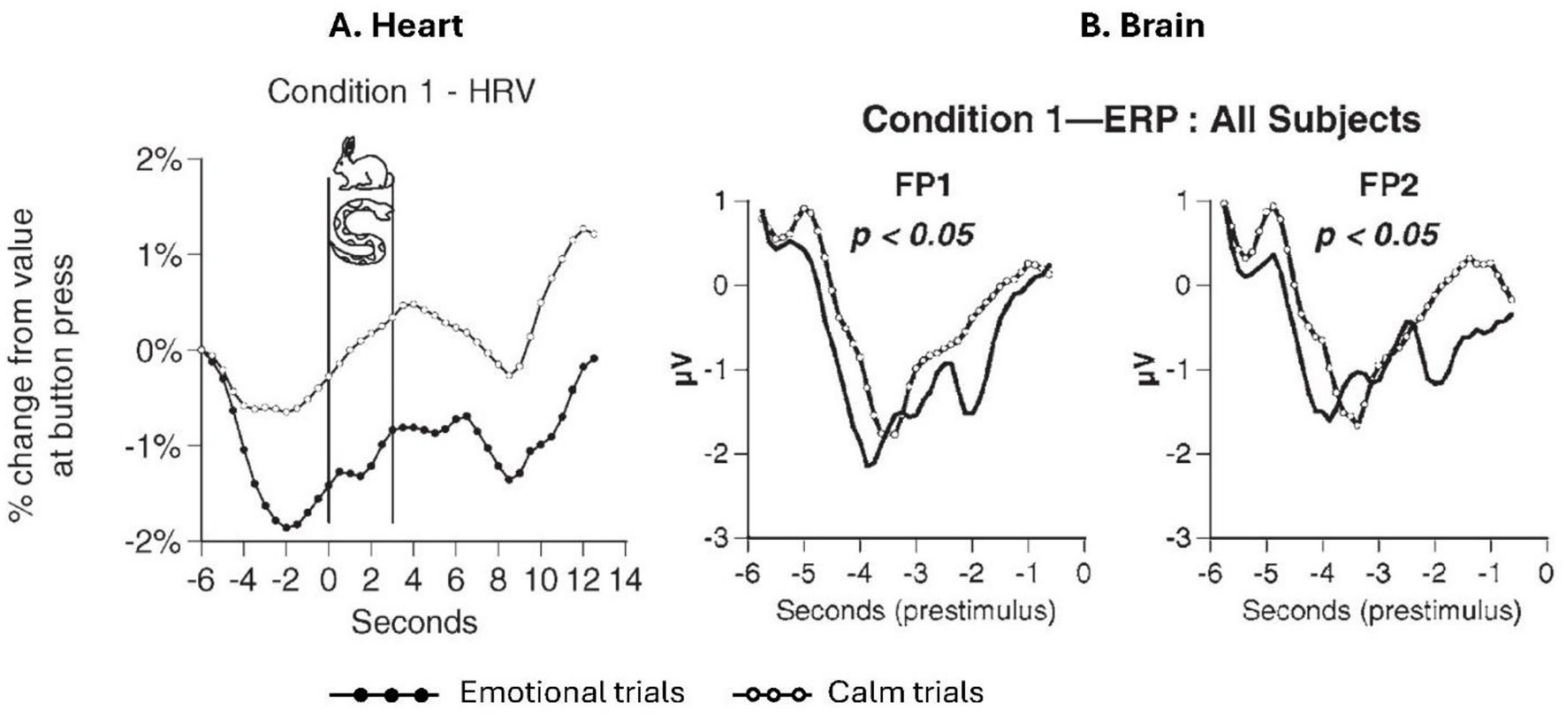
Heart and brain prestimulus response. **(A)** Shows the mean heart rate variability (HRV) response for the sample (*n*= 26) for “calm” vs. “emotional” trials, under experimental Condition 1 (baseline psychophysiologic mode). The “0” point on the “Seconds” timeline denotes stimulus onset. Significant differences (*p* < 0.01) in the pre-stimulus response to “calm” vs. “emotional” stimuli were observed, where the HRV curves for the “calm” and “emotional” photos begin to diverge—viz, ~4.5 s prior to the participants viewing the photos (adapted from McCraty et al., Fig. 4, 2004a, ©; used with permission). **(B)** Shows mean event-related potential (ERP) waveforms at FP1 (left frontal cortex) and FP2 (right frontal cortex) for the sample (*n*= 26) for “calm” vs. “emotional” trials. Data are shown for the 6-s pre-stimulus period for Condition 1; “0” seconds time point marks stimulus onset. There were significant differences in the overall waveforms at these sites (adapted from McCraty et al., Fig. 2, 2004b, ©; used with permission).

A ground-breaking Institute of HeartMath (IHM) experiment, in which RB was involved (McCraty et al., 2004a,b), augmented Radin's protocol with electrophysiological measures of both the brain and the heart response to pre-stimulus information—see Figure 1 for the experiment protocol.^1^ The study used a counter-balanced crossover design in which participants repeated the experiment after a two-week interval. All 26 participants (15 females and 11 males) were adepts in emotional management/spiritual practice techniques, with a decade or more experience at the time of the study. Random permutation analysis (RPA) was the primary statistical analysis method used to analyze the data.^2^ The major finding was that *both the brain and the heart received prestimulus information some 4–5 s before an “emotional” picture was randomly selected* for presentation to study participants, but not before a “calm” picture (Figure 2). In the heart, this was signaled by a significant heart rate deceleration in the heart rate variability (HRV) curve prior to the emotional stimulus (Figure 2A). This discovery of the heart's involvement has been corroborated in subsequent studies (Gillin et al., 2007; Tressoldi et al., 2009; Bradley et al., 2011; Rezaei et al., 2014), with one study finding that the heart's prestimulus response was 18 s in the future (McCraty and Atkinson, 2014).

Concerning the brain's involvement, under Condition 1—baseline mode, there were significant differences in cortical event-related potentials (ERPs), primarily in the frontopolar areas (FP1 and FP2; Figure 2B), with an increased negative-going wave and a faster onset of the positive-going wave in emotional trials. According to Karl Pribram, neuroscience advisor to the study, the negative-going ERP wave signals attentional processing, whereas the shift to a positive value marks the end of processing. Other regions of the brain were also involved—the left temporal area, occipital areas, and, to a lesser degree, parietal areas. In Condition 2—coherence mode, only one site generated a significant difference between emotional and calm trials, at the EEG midline site, Pz. For the prestimulus HBEPs, no significant differences were observed.

### Gender

Despite longstanding interest in the two different conceptions of intuition (viz, cognition-based and nonlocal) by quite distinct groups of researchers, there is a surprising scarcity of empirical studies on gender.^3^ One review of research on intuition in nursing, found “No evidence” of a gender differential (Holm and Severinsson, 2016). Yet, results from recent studies of decision making “styles” using questionnaires or interviews suggest that while men tend to be “brain/gut-based” and rely more on a “rational/logical” approach, women come more from the “heart” and prefer an “intuitive” style (Sinclair et al., 2010; Norris and Epstein, 2011; Fetterman and Robinson, 2013; Sinclair, 2020; Soosalu and Henwood, 2020). In a follow up experiment to studies using electrophysiological measures of brain activity (e.g., Bonnefond et al., 2014; Williams et al., 2019), Bao and colleagues augmented their design with behavioral measures of “intuitive” and “analytical” processing, reporting similar results to those just noted. Namely, a “female preference for intuitive thinking” in contrast to a male “preference for deliberative thinking” (Bao et al., 2022, p. 1). Moreover, there were differences in the brain regions involved—specifically, a parietal (P3b) component coupled with “stronger parietal alpha power activity” in women, compared to a frontal-central (N2) component in men.^4^

However, such approaches *cannot* capture nonlocal intuition. For that, an approach using objective physiological instrumentation is required which *must* include an unknown stimulus *and* measurement of the body's response during the prestimulus period—viz, *before* the stimulus is presented to research subjects. This enables measuring where and how in the body prestimulus information is received, and the temporal dynamics of sequence and function in the processing stream.

### Heart modulation of alpha and beta states

**Figure 3:**
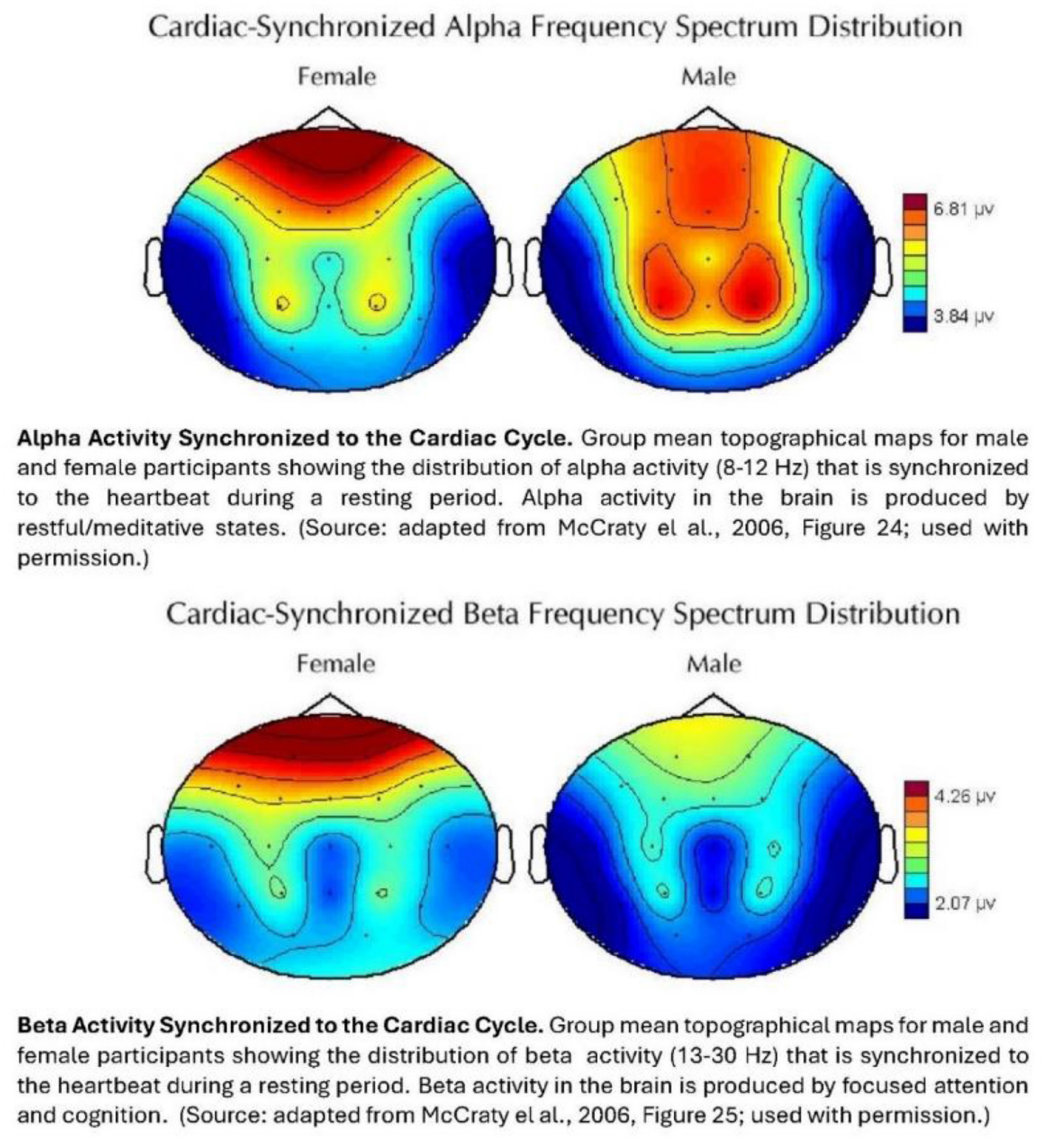
Synchronization of alpha and beta activity in the brain to the heartbeat.

But before we turn to this, it is informative to see how brain activity is mediated by heart activity during two “normal” states of consciousness (*not* during an intuition experiment)—viz, *alpha* (8–12 Hz) and *beta* (13–30 Hz), associated with restful/meditative states and focused attention/cognition, respectively. Investigating such “heart-brain synchronization” in other studies on the same population, IHM researchers found that a “significant amount of alpha rhythm [and lower frequency brain] activity is synchronized to the activity of the heart” (McCraty et al., 2006, p. 39).^5^ Moreover, as clearly apparent in the topographical maps (Figure 3), they found striking differences by gender. Whereas the females (*N* = 17) have more heart synchronized alpha activity in the frontal areas (*top* maps), in the males (*N* = 13) it spreads from the frontal cortex to the parietal region. In discussing alpha states, Stein writes:

This is the *starting place of matriarchal reality*, nonlinear and timeless thinking. The world is unified and flowing at this level, and this is the place of *daydreaming and creativity … sudden inspirations, intuitions and creative ideas, psychic healing, most forms of psychic receptivity* (Stein, 1988, p. 17; italics added).

What is notable is that *both* males and females, in the IHM study, are part of a population of longtime spiritual adepts, with a decade or more of training and practice in spiritual and emotional management disciplines. Yet, even so, a clear differential in alpha activity by gender is apparent.

The results for the beta waves show a distinctive pattern of synchronization to heart activity (*bottom* maps). Not only do the females have much more “background” beta activity than the males, but this is more synchronized to the heart in the *frontal* regions. As shown in the IHM intuition study, next, this pattern of significant heart-mediated brain activity in the frontal regions in females is *also* seen in their processing of prestimulus information about a future event.

#### Differences in prestimulus response

Returning to the IHM intuition experiment, differences in prestimulus response were revealed when the data were broken down by gender.^6^ For the *heart*, while females registered significant HRV prestimulus response to emotional vs. calm trials in both conditions 1 and 2 (*z pre* −2.66, *p* 0.004; *z pre* −2.26, *p* 0.01, respectively) this was observed for males only in Condition 1 (*z pre* −1.82, *p* 0.03). This suggests that females appear to be more attuned to prestimulus information from the heart, especially when they are in the self-induced state of psychophysiological coherence (Condition 2).

This finding of greater attunement in females to emotional prestimulus information from the heart in *both* experimental conditions is notable. Because *all* participants were *expert* practitioners of emotional management/spiritual techniques, such adeptness does not explain the significant HRV result for females in the coherence state. This suggests greater female sensitivity to emotionally charged prestimulus information from the heart.

**Figure 4:**
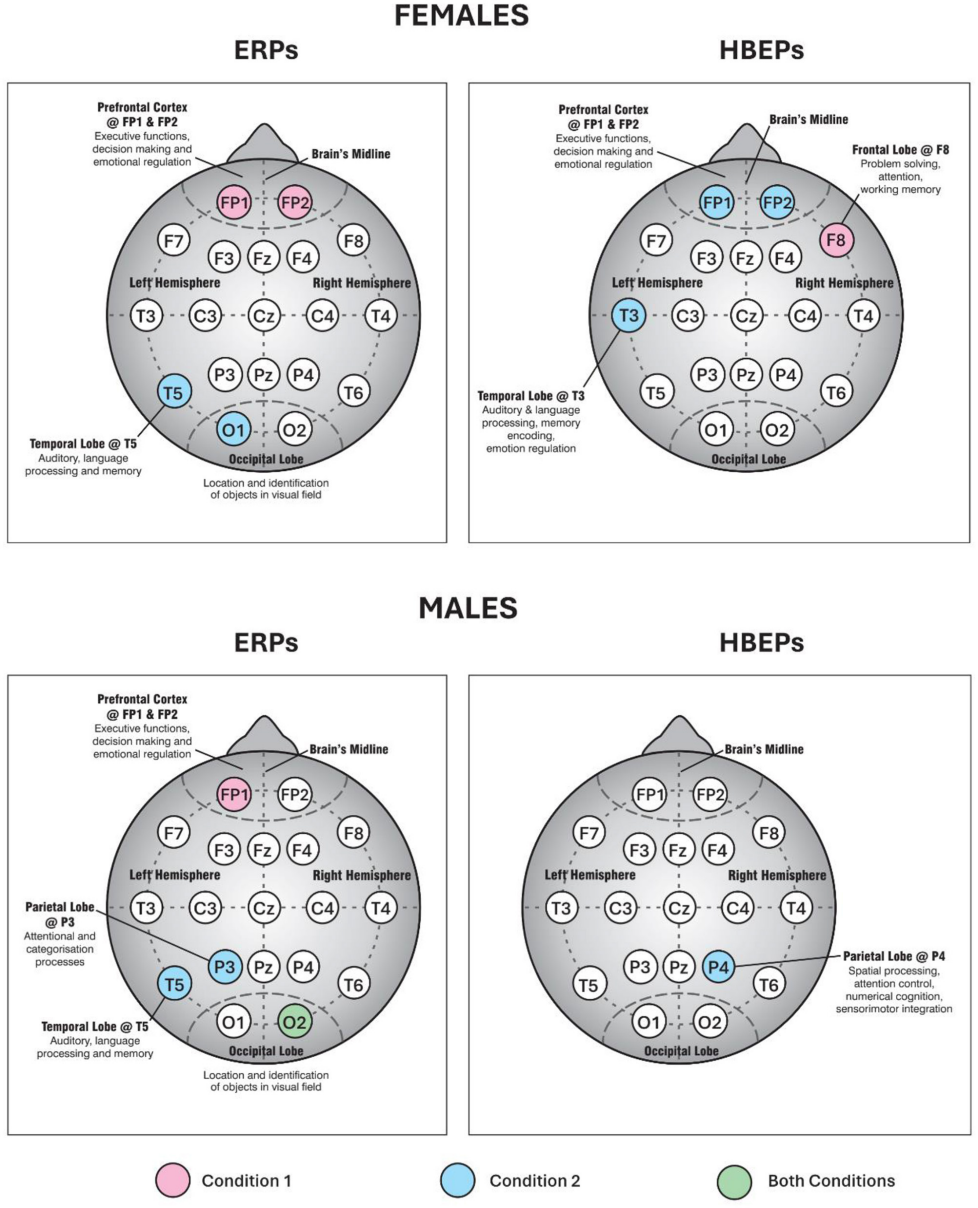
EEG electrode brain map showing prestimulus response by gender. The IHM study used a 19-node EEG electrode scalp net, as shown. The figure shows significant emotional vs. calm differences (*p* < 0.05) in prestimulus response in electrode locations for ERPs and HBEPs (except for FP1 in females ERPs— Condition 1, where *p* = 0.06, m.s.). In females, there is a concentration of prestimulus processing in the frontal cortex (FP1, FP2, and F8). In males, by contrast, except for FP1 for ERPs, processing activity is concentrated in the posterior regions (P3, P4, T5, and O2). (© Copyright 2024, R. T. Bradley and C. T. Torris; used with permission).

In terms of the *brain regions* processing the prestimulus information, there were gender differences in both the ERP and HBEP data (Figure 4). Overall, the most striking result is that whereas five electrode sites registered a significant emotional vs. calm prestimulus difference (*p* < 0.05) in Condition 1—baseline mode, nearly twice as many (9 sites) registered significant differences in Condition 2—coherence mode. For gender, there is a notable difference with the brain regions involved. In females, there is a concentration of prestimulus processing in the *frontal* cortex (FP1, FP2, and F8) which involve: executive/decision functions, emotional regulation; and attention/problem solving or working memory, respectively. In males, by contrast, except for FP1 for ERPs, processing activity is concentrated in the *rear* regions of the brain (P3, P4, T5, and O2) which involve: attention/categorization; attention/spatial/numerical cognition; sensorimotor integration/audition/language/memory; and visual perception of objects, respectively.

Overall, two conclusions are suggested. First, for men, the results indicate that the prestimulus information is processed *dispassionately* by the brain for *attentional significance*. By contrast, in women, it is the *emotional significance* of the future event that is being *evaluated* by the brain for *potential action*. The second conclusion is that Condition 2—coherence mode, was most associated with significant prestimulus brain response, especially in females for both ERPs and HBEPs. In males, with one exception for HBEPs (P4), this was confined to ERPs.

**Figure 5:**
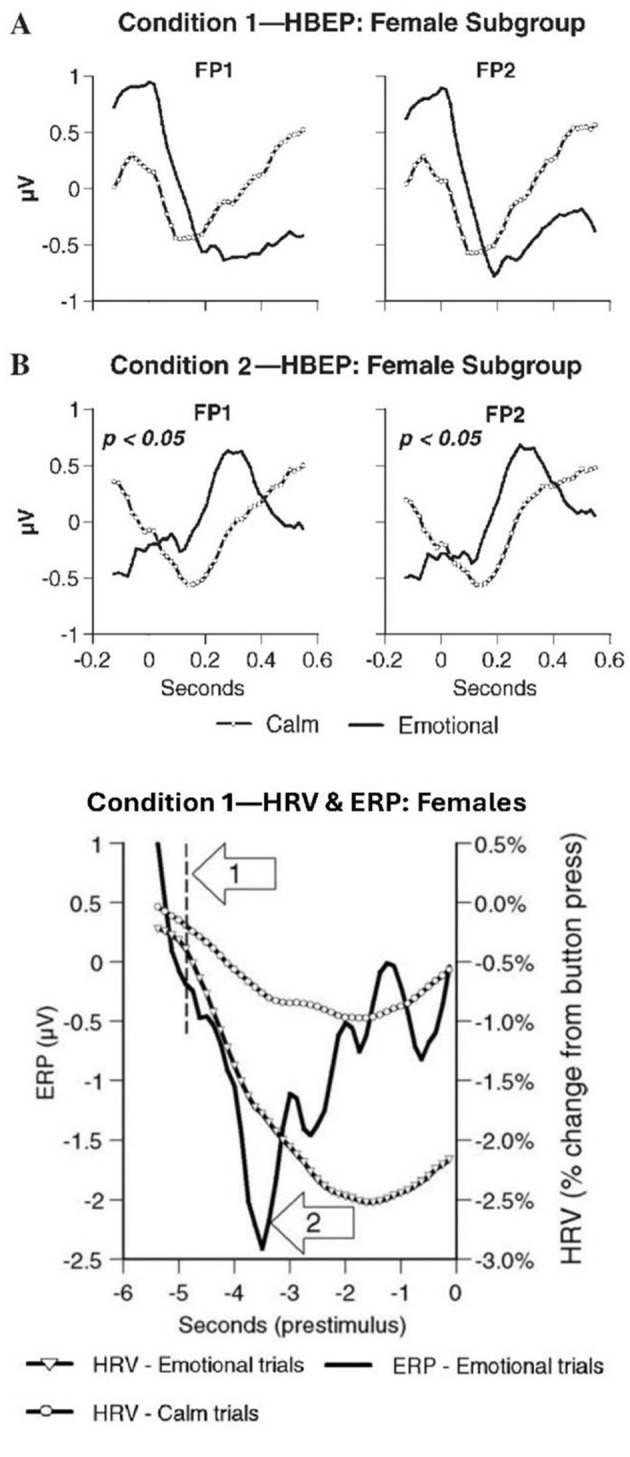
Prestimulus results for females. **(A)** Heart to brain prestimulus input. Mean prestimulus heartbeat-evoked potential (HBEP) waveforms at FP1 and FP2 for the female subgroup (*n*= 15) for “calm” vs. “emotional” trials. Data are shown for Condition 1 (A) (baseline psychophysiological mode) and Condition 2 (B) (post physiological coherence), with the “0” time point denoting the peak of the ECG R-wave. In Condition 2, there were significant differences in the HBEP waveforms for “calm” vs. “emotional” trials at both frontal sites. Note the marked differences in the morphology of the waveforms in Condition 2 compared to Condition 1. It is noteworthy that the ERP analysis for the female subgroup revealed significant differences at FP1 and FP2 in Condition 1, but not in Condition 2 (see Figure 3, original article), which suggests that information processing in the frontal cortex was modified by afferent input from the heart (adapted from McCraty et al., Fig. 4, 2004b, ©; used with permission). **(B)** Temporal dynamics of heart and brain prestimulus response. This overlay plot shows the mean event-related potential (ERP) at FP2 (in the brain) and heart rate (HRV) deceleration curves for the female subgroup (*n*= 15) in Condition 1 during the prestimulus period. (The “0” time point denotes stimulus onset.) The heart rate deceleration curve for the “emotional” trials diverged from that of the “calm” trials (sharp downward shift) ~4.8 s prior to the stimulus (arrow 1), while the emotional trial ERP showed a sharp positive shift ~3.5 s prior to the stimulus (arrow 2). This positive shift in the ERP indicates when the brain “knew” the nature of the future stimulus. The time difference between these two events suggests that the heart received the intuitive information about 1.3 s before the brain (adapted from McCraty et al., Fig. 5, 2004b, ©; used with permission).

For females, there were other notable results (Figure 5), which are worth recounting in detail for they reveal the *dynamics* of the heart's role in modulating the brain's response to prestimulus input. We begin with the HBEPs ^7^ (Figure 5A).

In condition 1 [baseline—“normal” function; Figure 5A, top 2 graphs], although the females' HBEPs were not significantly different in the frontal areas (FP1 and FP2), their emotional trial HBEP waveforms start at a more positive level and shift sharply negative. This negative shift in the HBEP waveforms adds to the overall negativity of the emotional trial ERPs, which were significantly more negative than the calm trial ERPs in condition 1.

In condition 2 [coherence mode; bottom 2 graphs], however, a quite different picture emerges. In the coherent condition, the females' emotional trial ERPs did not have the increased negativity and were therefore not significantly different, but their HBEPs were significantly different. In this condition, the HBEPs start at a more negative level and shift sharply positive, apparently reducing the overall negativity of the ERPs …. Thus, it would appear that *afferent input from the heart plays an important role in modulating activity in the frontal cortex* (McCraty et al., 2004b, p. 333–334; italics added).

**Figure 6:**
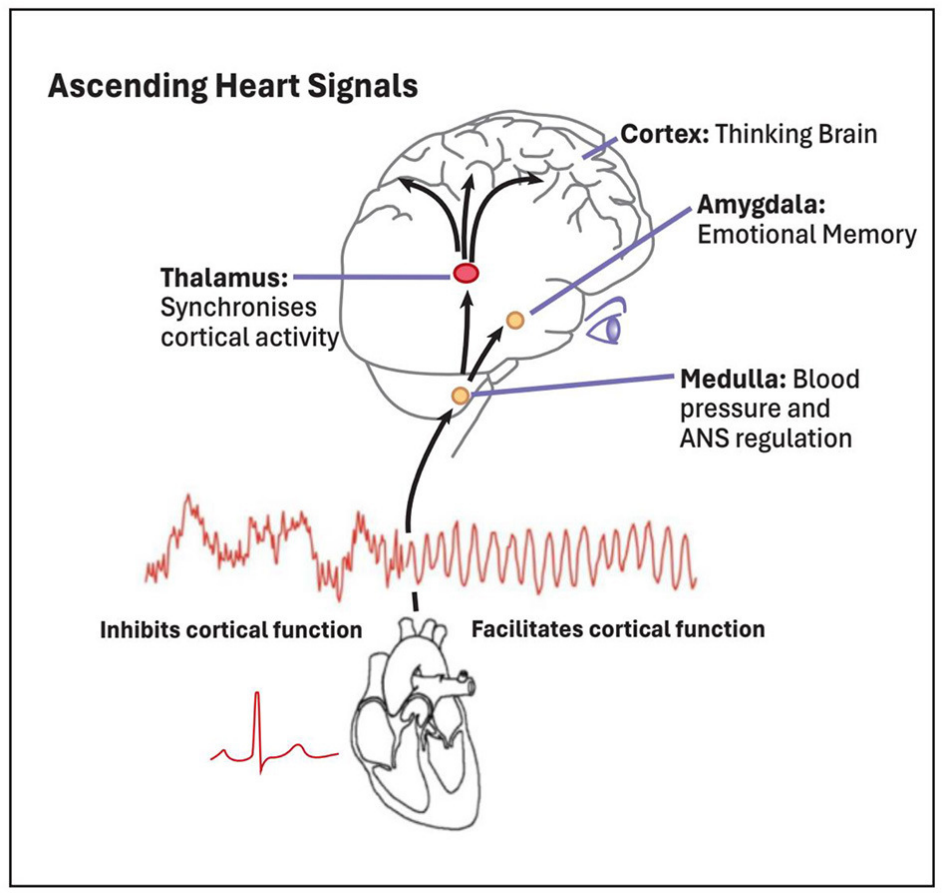
The heart modulates emotional processing in the brain via afferent pathways. Illustrated are the afferent (ascending) pathways by which signals generated by the heart are transmitted to key centers in the brain exerting a continuous impact. These heart signals not only impact autonomic regulatory centers in the brain (e.g., the medulla, which regulates the ANS and blood pressure), but also cascade up to higher brain centers involved in emotional and cognitive processing, including the thalamus, amygdala, and cortex. As shown, during emotional stress, patterns of heart activity are erratic and disordered, producing an *inhibition* of higher cognitive and emotional functions. In contrast, during positive emotions heart rhythm activity is more ordered and stable [sine wave-like], and this pattern of input to the brain *facilitates* cognitive function and reinforces positive feelings of emotional stability and wellbeing (adapted and redrawn from Bradley et al., 2007, Figure 11.4, ©; used with permission.

This is entirely consistent with the well documented role the heart plays, via afferent pathways, modulating emotional input to the brain, as depicted in Figure 6 (Tiller et al., 1996; McCraty et al., 2006).

#### Processing dynamics

An “overlay” plot of the HRV data with the ERP data for females in Condition 1 (baseline) during the prestimulus period (Figure 5B), sheds light on the temporal dynamics of processing intuitive information by the brain and heart. If we examine the two HRV curves, “arrow 1” marks the point at which the heart rate deceleration curve for “emotional” trials diverges sharply down from the curve of the “calm” trials. This occurs ~4.8 s prior to stimulus selection and presentation (marked by “0” on the “Seconds” timeline). Turning to the ERP curve for emotional trials, it is sharply negative (downward) during the first second and-a-half of the prestimulus period, but then makes a sharp positive shift (upward)—“arrow 2”— ~3.5 s prior to the stimulus. “This positive shift in the ERP indicates *when* the brain “knew” the *nature* of the future stimulus. The time difference between these two events suggests that the *heart received the intuitive information about 1.3 s* ***before*** *the brain*” (McCraty et al., 2004b, p. 332; italics and bold added). In short, the heart receives information about the future event first, which it transmits to the brain to weigh the need for potential action. Pribram explains it this way:

… the negative-going [ERP] wave is indicative of attentional processes—“the brain says to itself: “I have to pay attention, stay alert, and process this information.” The positive shift in the ERP wave marks the end of processing—*the brain acknowledges: “The information is here, I have to do something* (McCraty et al., 2004b, p. 332; italics added).

Given the *kind* of stimulus the study used to arouse emotion (pictures of menacing wild animals, threatening situations, etc.), it seems clear for female participants—but less so for males—that this evoked a strong *negative* emotion: fear of danger or threat. Thus, in females, the brain is assessing the prestimulus information for *adverse* risk or harm. In real life, this would give a woman a few seconds of intuitive forewarning for evasive/protective action—to *avoid* danger. By contrast, the males dispassionate attentional focus seems a response to face a prospective danger “head on.” Such differences appear reflective of a deeper bio-genetic difference: in females, for a procreative, protective role as “mothers” and “caregivers;” in males, for a guardian, defender role as “fathers” and “warriors”.

### Two Propositions

Our research review suggests two propositions.

*Proposition 1. All women are endowed with psychic proficiency*.^8^

With few exceptions (e.g., Holm and Severinsson, 2016), this emerges as the primary empirical generalization. This psychic proficiency, encoded into women's bio-emotional and spiritual makeup, emerges from the existential implications of procreation—viz, of females having, raising, and protecting their offspring and the familial group. Maternal “intuition”—being attuned to and sensing an offspring's needs and wellbeing—even at a distance, is a female “skill” acknowledged by all cultures (Gardner et al., 2020). This is the basis of women's intuition, and, as documented, it is undergirded by differences in heightened socioemotional sensitivity, in brain response and activity, and in heart attunement and response—*especially* in sensitivity to and the processing of *prestimulus* information. But this psychic potential requires practice and experience to develop as a reliable, efficacious skill.

*Proposition 2. All women have the capacity for selfless/unconditional love*.

This capability, too, is encoded into women. Given the challenges, stresses, and uncertainty involved, unconditional love is a necessity for having and successfully raising children. As CT notes from personal experience as a “mother,” “Despite the discomfort of giving birth and the accompanying commonly experienced thought during labor—“I'll never do this again!”—that first cry from my child opened an enormous well of ecstatic love, happiness, and affection making it very easy to repeat the experience.” The newborn infant's needs, development, and well-being requires a “good enough” mother capable of *attuning* herself through a selfless bond of unconditional love to the infant (Parsons et al., 2010): being able to sense the often implicit signals an infant or child is communicating. Without such bio-socioemotional attunement—the basis of Stein's “psychic link”—the infant's care, development, and wellbeing are compromised, which can result in psycho-social pathologies for the child or adult in later life (Schore, 2003). In short, successful motherhood requires *selfless/unconditional love*. Such love is foundational for mother's intuition *and* for development of a mutual psychic bond between mother and child.

### Summary

A century of research documents that nonlocal intuition is an inherent psychic proficiency possessed by *all* humans. Specifically, that the psychophysiological system can accurately perceive information from a distant or future source, and that this result *cannot* be explained by researcher bias, the different methods used, research artifacts, or by chance (Radin, 1997a). Moreover, studies of the sensory perceptual channels involved—visual, auditory, the “gut,” the heart, and so forth^9^—rule out a so-called psychic “sixth” sense. Instead, they show that *pre*-stimulus information is received and processed through the *same* psychophysiological systems as “normal” sensory input. Of importance, from the presentiment experiments, is that the *body's psychophysiological system typically responds to a future emotionally arousing stimulus some four to seven seconds before the stimulus is presented*.

The IHM study showed that *both* the brain and the heart were involved—with the heart receiving the prestimulus information a second or so *before* the brain. The study also found that women are more attuned to the prestimulus information from the heart, which is evaluated in the brain's frontal cortex for the emotional significance of the future event. By contrast, not only are men somewhat less attuned to prestimulus input from the heart, but this information is processed by the brain dispassionately with respect to its attentional significance, mainly in the left hemisphere rear region. However, due to the small size of the gender samples in these studies, replications are urgently needed to validate the generality of these findings.

## Mother-offspring intuition

While these results shed light on the questions of *where, when*, and *how* nonlocal intuition information is processed in the body, there remains the longstanding enigma of mother-offspring intuition, noted at the outset.

A potential biological mechanism—*microchimerism* (the presence of a low-level allogeneic cell population within a host body)—was identified in a posthumous study of the brains of 59 women (Chan et al., 2012). Thirty-seven (63%) had traces of male DNA (Y chromosome) in several regions of their brains. But the Y chromosome did not come from the women's fathers because the women would have been born male if that was its source. Logically, therefore, the male DNA in the women's brains must have come from their sons. However, since the examination was posthumous, it was not possible to ascertain which, if any, of the 37 women had experienced mother-offspring intuition in relation to an offspring during their lifetimes.

### Mother's intuition

“Mother's intuition” is a colloquial concept, across cultures (Gardner et al., 2020), referring to a mother's tacit sensing of her child's needs or of an imminent threat. To respond “effectively” to her newborn infant's endless cascade of urgent needs—which often leave no time for a deliberative approach, the “good enough” mother quickly learns to trust and hone her inner sense of what to do (Parsons et al., 2010). Known as “intuitive parenting,” this proficiency is regarded as requisite for raising a child (Papousek and Papousek, 1987; Parsons et al., 2010). Indeed, when blocked or impeded by a mother distracted by life circumstances (such as acute depression), adverse consequences are typical for the infant's care and development (Maté, 1999; Bjertrup et al., 2019; Schore, 2003)—especially, attachment bonding.^10^

By the time the offspring has reached adolescence, this relation has often become a *two-way* psychic bond where *either* can instantly intuit something amiss or that harm/danger is about to happen to the other. Accordingly, we broaden the concept of mother-offspring intuition to that of a mutual psychic connection between the pair, whether proximate or separated in distant locations—even continents apart. To understand how this tacit bond of “inseparability” is created, we postulate that it is initiated at conception as a nonlocal connection of *multiscale entanglement*, as described below.

### An example^11^

CT describes her experience of “mother-offspring” intuition with her eldest son, Aaron.^12^

My son, Aaron, has always exhibited strong intuition and sensitivity to what was happening with those he loved. Growing up, Aaron was known to be the person to go to, to find lost articles. He had an uncanny “intuitive” sense of where they were. As an adult, Aaron moved to another state which made it harder to connect with each other. But I never worried about our relationship as those times when we connected, our bond of love and intuitive connection was real and always present. This was illustrated with what happened in 2007. I was going through the breakup of my 20-year marriage. I was trying hard to live life in a “normal” fashion, not giving myself access to my feelings.

One afternoon, driving through the mountains, something happened that opened the gate to my feelings. I pulled over, sobbing, on the side of the road. Within seconds my cell phone rang. Aaron was calling, asking “Mom, are you alright?” Crying hard, I answered “No! I am so glad you called!” I explained what was happening. Aaron comforted me, told me he loved me, and was sorry to hear what I was going through. I had not spoken with Aaron for several years, yet he felt my distress and followed his intuition to check in with me. He provided exactly what I needed at that time.

Several years later, in 2015, I met my soulmate (a New Zealander) and we married in 2016. Early in 2020, I found myself in my New Zealand home at the time of COVID 19 lockdowns. I worried about my children in the US and prayed for their good health and safety. I was able to speak with my other children but had no way to reach Aaron. I wished I had more contact with him during this uncertain time. I wanted him to feel my love and support but had no way to reach him. I decided to begin Metta (LovingKindness) Meditation (Salzberg and Goldstein, 2001) which is performed by holding an image in mind while sending loving intentions. Thus every morning I sent him my unconditional love and support. I knew that he would receive my psychic “message”.

On the third day of meditation, Aaron called, and we had a long and wonderful conversation. I have no doubt that the love I sent Aaron through meditation, was received by him through our frequency attunement. I placed no expectations for any outcome other than that he would, on some level, feel my love for him. To my great delight, it inspired him to call, and we have been in close communication since then.

On the assumption that mother-offspring experiences of nonlocal intuition are valid, we are faced with a fundamental question: *how* is an “ever-present, always ready” tacit bond for instant communication established between a mother and her offspring?

## Creation entanglement

To develop the concept of multiscale entanglement we begin with an overview of the evidence of quantum entanglement.

### Quantum entanglement

Instant *nonlocal* connection irrespective of distance, is known as *entanglement* in quantum physics, and, following a breakthrough experiment design (Clauser et al., 1969), has been repeatedly verified in experiments since the 1970′s (Aczel, 2001).^13^ Initially demonstrated in a laboratory between a pair of entangled particles separated by a few meters (Freedman and Clauser, 1972), then documented across hundreds of meters—implicating a signal velocity greater than the speed of light (Aspect et al., 1981), entanglement was given its strongest confirmation in an audacious experiment, by Gisin and his collaborators, between two locations linked by a fiber-optical cable 16 km (10 miles) long (Tittel et al., 1998). In his book, *Entanglement: The Greatest Mystery in Physics*, (Aczel 2001) points out that “Because of the experimental setup, a signal from one end of the cable to the other, telling one photon what setting the other photon found, would have to travel at *ten million times the speed of light*” (page 237; original italics). Conclusive “proof” of the reality of entanglement came from experiments using a more complex three-particle system (Zeilinger et al., 1997). The results decisively ruled out Einstein's “local causality” and Bohm's “hidden variables” hypotheses—both derived from their (and others) belief of the “incompleteness” of quantum theory. Furthermore, entanglement has been found at the *macro scale* between a pair of atoms (Hofmann et al., 2012)—including when separated by a few hundred meters (Rosenfeld, undated; van Leent et al., 2022),^14^ and in larger molecular structures (Zeilinger et al., 1997).^15^ Finally, atom-subatomic particle entanglement was recently documented across the macro-micro scale “divide” (van Leent et al., 2020). In short, these days entanglement is accepted as “scientific fact”—a status formally acknowledged with the awarding of the 2022 Nobel Prize in physics to John Clauser, Alain Aspect, and Anton Zeilinger for their pioneering discoveries.^16^

In these experiments, the particles are “created” as an entity—viz, brought into “existence” at the same instant in a laboratory under controlled conditions, and isolated. When spatially separated from each other by a vast distance (in quantum terms), and one particle is subjected to an experimental stimulus (e.g., a change in “spin” direction), the other is observed to *immediately* reverse its “spin.” Thus, because the individual particles respond as if they are a single, *inseparable* unit, physicists describe them as *entangled*. Indeed, this behavior has led some physicists to characterize the relation between entangled particles as “passion at-a-distance” (Shimony, 1993, p. 135).

### Multiscale entanglement

Building upon these discoveries, RB has developed the concept of *multiscale entanglement* to describe how a channel of instant nonlocal communication is created between macroscale objects by entanglement among the atomic and subatomic constituents involved through the multiscale processes that generate emergent order (Bradley, 2024c).^17^ This can be generated in three ways: through the micro-macro scale bonding processes involved in the creation of new life, through the bonding processes in a relationship of selfless love–that Tiller describes as “human-human entanglement” (see Tiller et al. (2005), p. 207–210), or through selfless mutual bonding in shared experience of events of heightened emotional significance.^18^ As described momentarily, energetic entrainment between/among the hearts involved is posited to mediate the process.

Energy emissions from subatomic particles within objects at the molecular level and above are *coherent*, reflecting the endogenous constraints of the object's macroscale order. This well-documented property of micro-macro scale bonding in emergent systems is known as *quantum coherence*,^19^ and is *not* found for isolated particles which emit energy radiation at random. Insofar as multiscale entanglement is consistent with the micro-macroscale bonding in emergent systems, quantum coherence is an expected property.

**Figure 7:**
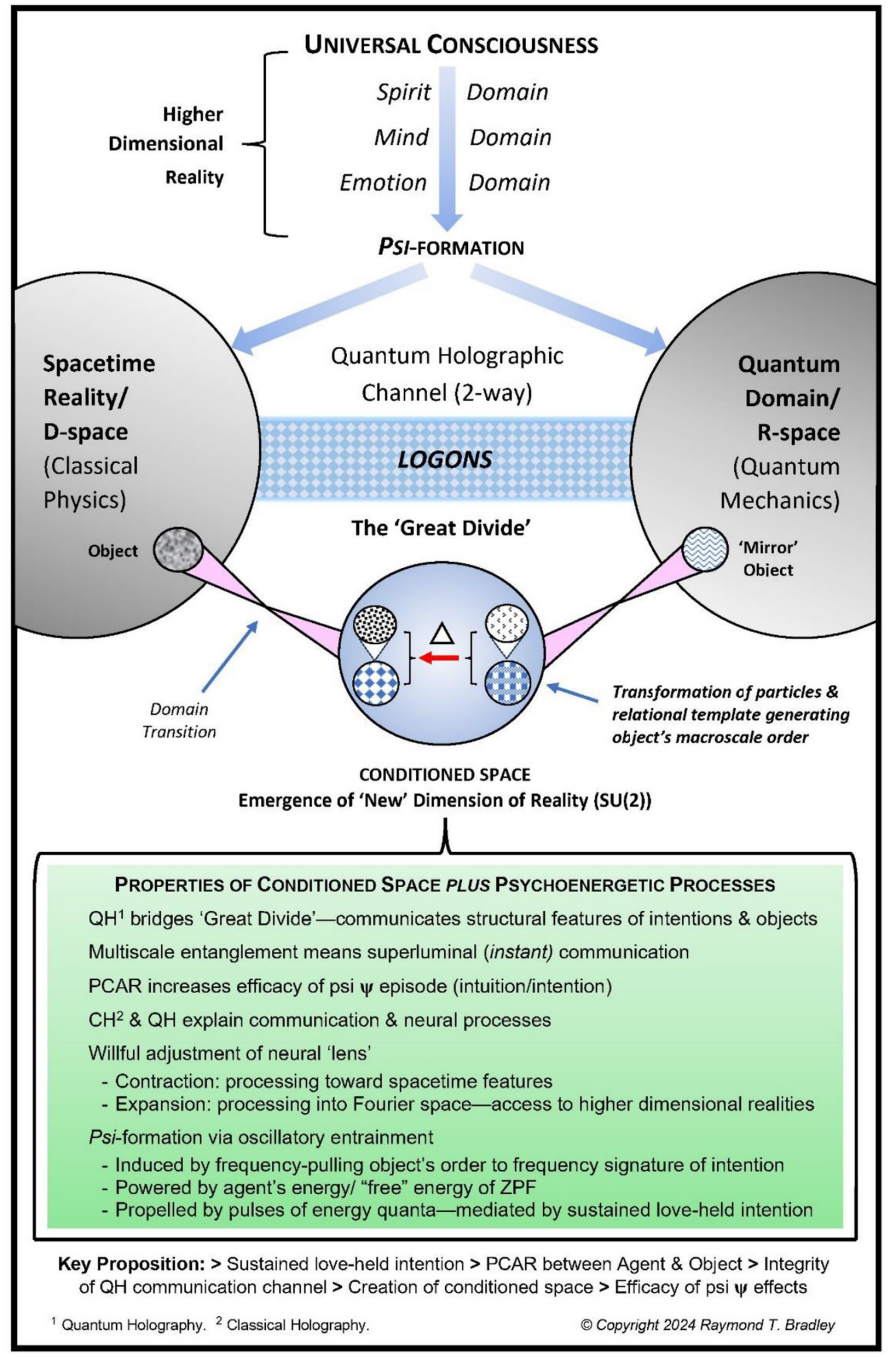
Quantum holographic revamp of Tiller's model. Depicted is RB's rework of Tiller's model which shows the quantum holographic logon channel (the “bridge”) between spacetime reality and the quantum domain. In the psychoenergetic process of *psi*-formation, the Agent's intention and energy is simultaneously transmitted though the logon channel instantly, via multiscale entanglement, to the target object in spacetime and its “mirror” in the quantum vacuum. Sustained love-held intention, mediated by PCAR, generates domain transitions to Tiller's “conditioned space” and the emergence of a “new” dimension of reality [SU(2)]—Tiller's “higher gauge symmetry state.” It is here that the object's particles and its relational template (RB's addition) are transformed, thereby creating the will-induced macroscale change of the object, as described in (Bradley 2024c) (from Bradley, 2024c, © copyright R.T. Bradley, 2024; reproduced with permission).

Before moving on, a fundamental difference between these two concepts of entanglement must be noted. Quantum physics views all matter, entities, force fields, and energy at this level of reality as *inert*—viz, that life, sentience, and agency are *not* involved.^20^ By comparison, multiscale entanglement is a broader concept encompassing the microscale of quantum phenomena, the macroscale domain of biophysiological reality, *and* the higher dimensional spaces of psychosocial and psychic reality. This difference in scope has necessitated the development of a larger reference frame (see Figure 7, below) that incorporates these aspects of reality to adequately address human nonlocal communication—of which mother-offspring intuition is one facet.

The creation of new life produces a bond of inseparability by *multiscale entanglement* of the constituent atoms and subatomic particles involved. The process is induced by the mother's unconditional love for her “baby”, and thus is mediated by her sustained state of heart coherence, as described momentarily. Initiated at conception, the joining of a sperm with an ovum creates “cellular union” between the two cells in which multiscale entanglement, via harmonic resonance, creates a bond of inseparability across the macro (atomic) and micro (subatomic) constituents of the zygote (fertilized ovum). During the gestational processes of embryonic development, the process continues—encompassing all molecules, cells, organs, and so forth—and creates a bond of energetic “union” within the fetus, across all levels of scale. This process expands and entangles the fetus with the mother, via their atomic and subatomic constituents. Driven by the more powerful, dominant rhythmic energy of the mother's heart, the fetus' system is frequency-pulled across all levels of scale into harmonic resonance with the mother's systems. The result, by birth, is the creation of a bond of nonlocal inseparability between them. After birth, insofar as the mother maintains a mostly persistent state of unconditional love for her child, a two-way bond of psychic connection develops by adolescence.

This is an entrainment process mediated by the mother's heart—the strongest generator of rhythmic energy in the two bodies (Armour, 2003). Sustained states of selfless/unconditional love^21^ frequency-pulls the fetus' heart activity, via harmonic resonance, to induce a phase shift to the rhythm of the mother's coherent pattern which is imprinted on the fetus' psychophysiological system. Such “phase synchronization” between the two cardiac systems was documented by Van Leeuwen et al. (2009). They used techniques from nonlinear dynamics to reveal a previously undocumented “phase synchronization” between the heartbeat patterns of the mother and fetus, which Ivanov et al. (2009) describe as a “marker of *coupling* between their autonomous cardiac systems” (italics added). This “coupling” between hearts creates a coherent channel of connection through which energetically encoded information is communicated, including that involved in regulating the fetus' neural development via “heart-brain synchronization” between the pair.^22^ Two likely information processing mechanisms—“fractal self-similar resonance”^23^ (Marks-Tarlow, 2020) and quantum holography (Bradley, 2024c)—are described below. Heart rhythm synchronization is well-documented between humans (McCraty et al., 2006; Feldman et al., 2011; McCraty and Atkinson, 2014), between humans and their pets (Sheldrake, 1999; Bradley, 2012), and among animals (Sheldrake, 1999) and, as noted above, is directly implicated in psychic communication.

To the extent that the mother's emotional state is mostly positive, multiscale entanglement between the two hearts creates a bond of inseparability, and hence the energetic channel for instant tacit communication. Insofar as the mother wants her pregnancy and loves the fetus, multiscale entanglement will survive stressful life or socioemotional circumstances, as illustrated with one of CT's clients, momentarily.

However, if the pregnancy is *unwanted* and the mother maintains a resentful attitude or worse toward the fetus, her persistent negative emotions generate an erratic, *incoherent* heart pattern of energy which impedes multiscale entanglement and, hence, the potential for a bond of nonlocal connection between them.^24^ Further, the fetus will be imprinted, via heart-to-heart entrainment, and born with the same negative emotional setpoint as the mother, suffering a cascade of psychopathological consequences in later life (Maté, 1999; Schore, 1994, 2003).^25^

### A case of entanglement: Ella's story

Ella contacted me (CT) for therapy, seeking help for “overwhelming anxiety and panic”. Ella was a 34-year-old woman, still living with her parents. Ella appeared nervous and vulnerable. She said it was hard for her to come to my office, as she was so fearful of leaving her parents' home.

During Ella's evaluation, she reported feeling anxious from childhood but did not know why. Ella reported normal childhood and teen years, with no incidences of trauma. She described her parents as loving and protective, stating she had never had “sleepovers” at friends' homes, but rather, friends would stay with her. Ella had no history of substance abuse, sexual abuse or serious medical or accidental traumas. Ella stated her anxiety increased when she started college, though she still lived with her parents. Ella reported having positive relationships with all family members and her boyfriend of 3 years, who would visit her at her parents' home. Yet Ella was paralyzed with *fear that if she left her parents' home her mother might die* while she was away. This made it impossible for her to leave. Ella graduated from college (10 years previously) but had never worked a job, due to this fear. Ella's expressed goal was to lead a normal life, to spend nights with her boyfriend at his place, to travel, to be able to work and to have her own apartment.

Various interventions were tried, simple goals were set, to no avail. Labs and medical exams were normal. Ella used no recreational drugs or prescriptions. After a month of working with Ella, I found no successful method for understanding or decreasing her anxiety.

During this time RB was working on his book, *The Lens of Love*, and I remembered his chapter on entanglement and the mother-fetus bond. Could it be that Ella had developed an emotional set-point of “anxiety” that was imprinted in the womb by entrainment with her mother's psychophysiological systems? And that the specific “fear of death” in her mother's consciousness was implicitly conveyed via the psychic bond between them?

I asked Ella “do you know anything about your mother's emotions or life situation when she was pregnant with you? Ella answered “yes!” Ella explained her mother experienced a devastating miscarriage before Ella's conception. Ella's mother feared she would miscarry again, or that Ella would die after birth. Ella further explained as a young child, her mother was vigilant and very protective of her—always “watching out for my safety.” Ella said this continued throughout her school years, only easing when Ella started college.

I felt I had discovered the basis of Ella's anxiety and her fear of leaving her mother. I explained to Ella that her mother's anxiety during pregnancy had likely been transferred to Ella as a fetus, creating an unconscious emotional setpoint for a baseline state of anxiety, operative from birth. Whenever Ella was under stress, her mother's specific “fear of death” was activated in Ella's consciousness through the psychic bond between them. I told Ella “Now that we know, *you can change this*! You *don't* have to carry that trauma for the rest of your life!”^26^

From that point on, Ella progressed on her goals of “normalcy.” She went on dates with her boyfriend, leading up to spending a night with him, and even planned a trip with him to visit another country. Knowledge and understanding are empowering tools for change!

### Other ramifications^27^

These prenatal dynamics and the mother's life circumstance and socioemotional experience produce a similar potential for twin fetuses—viz, the creation of multiscale entanglement and a bond of psychic communication between/among them. While involving a different mechanism—namely, unconditional love, the trauma of rejection and separation from its biological mother suffered by an orphan can be mitigated by loving adoptive parents. By the same token, interactions beyond the family circle based in selfless/unconditional love have a similar potential to create multiscale entanglements, and thus psychic connection between/among those involved.

### A channel for epigenetics?

While outside the focus of this work, multiscale entanglement may shed light on the mechanisms involved in transgenerational trauma. At present, research shows that the primary mechanism is *epigenetic*—viz, that the originating trauma affects the *chemical marker* for a gene (rather than the gene itself) or other “epigenetic pathways,” and that it is this “change” that is communicated across generations (e.g., Costa et al., 2018; Gapp et al., 2020; Yehuda, 2022).^28^ However, transgenerational trauma may be accompanied by *emotions* and *imagery* which do not seem sourced from an individual's own life, but appear to originate from trauma experienced by a family member of an earlier generation. This suggests that transgenerational trauma may involve more than a bio-chemical mechanism. Specifically, that a *psychoenergetic channel, via multiscale entanglement, tacitly communicates the emotional experience and imagery of trauma suffered by members of older generations to family members of succeeding generations*. This is illustrated in a thumbnail sketch of another of CT's cases.

### Transgenerational trauma: Joseph's story

Joseph (72), sought therapy for *acute depression, anxiety, shame, guilt, and debilitating nightmares*, stating “*I've always been depressed—I am broken beyond repair!*”

Joseph reported multiple historical traumas: at 12, his own serious illness followed by father's death; at 27, a debilitating motorcycle accident; and at 60, his ex-wife's cancer death. He blamed himself for these traumas, though in reality he was innocent.

While talk therapy and various healing modalities helped, Joseph would show initial improvement but then quickly regress. The nightmares were puzzling. Joseph was troubled because he could not remember them, feeling they

were important. My therapist's intuition told me *I was missing something*.

Digging deeper, vital aspects of family history were revealed. Joseph's father was born in Germany. Joseph's father and family were devoted practitioners of Judaism before WW II. His father was able to obtain visas only for himself and one sibling to migrate to USA. *All other family members perished in the Holocaust*. Joseph was born in the USA and did not consciously relate to his Jewish roots—“*We never practiced that.”* A novel hypothesis came to mind: *Joseph was suffering “Transgenerational Trauma”*—also known as *Epigenetics*. Joseph's symptoms mirrored those found in Transgenerational Trauma and Survivor's Guilt. This could explain Joseph's reported symptoms.

As this information was revealed and understood, Joseph began to heal. Through grief rituals, meditation, and self-empowerment practices, Joseph's depression lifted, his nightmares disappeared, and Joseph was able to finally move forward with his life.

### Summary

Building on quantum entanglement, the concept of multiscale entanglement involves two-way micro to macroscale entanglement between a mother and a fetus, created at conception. It evolves during gestation by entanglement among subatomic and atomic constituents of cells, extending to molecules, organs and so forth within their bodies. After birth, this entanglement is postulated to provide a lifelong nonlocal bond of inseparability between the pair. By adolescence, this bond creates an ever-present, always ready psychic channel for instant nonlocal intuition between a mother and her offspring, we illustrated with examples from CT's experience with her adult son, Aaron.

If a “loving” mother suffers persistent emotional distress/trauma during gestation (e.g., Ella's mother's fear of another miscarriage while pregnant with Ella), this experience is transmitted, via multiscale entanglement, to the fetus. The trauma is tacitly experienced by the latter, in adolescence or adulthood, as an inherent, unexplainable anxiety/panic. When the offspring is triggered by stress, the imagery of the mother's trauma experience is psychically activated, as illustrated by Ella's fear of her mother's death if she ventured away from home^29^.

An implication of the concept for transgenerational trauma is that in addition to a bio-chemical *epigenetic* mechanism, multiscale entanglement creates a *psychoenergetic channel* for tacit communication of the emotions and imagery of the trauma experience to family members of succeeding generations. This was briefly illustrated in Joseph's suffering transgenerational trauma from his father's horrific “Sophie's choice” dilemma of being able to save only one family member and the others' terrifying end in the Holocaust.

In essence, the basic proposition is that multiscale entanglement creates a two-way psychic channel for instant tacit communication between the mother-offspring pair. The all-important question is *how*: *what are the processes involved?*

## A new psychophysics

Drawing heavily on RB's recent work (Bradley, 2023, 2024b,c), a new approach to explaining psychic phenomena is sketched in what follows. We begin with William Tiller's reconceptualization of physics, which is foundational.

### William Tiller

Grounded in the discoveries of four decades of experiments documenting the effects of “lovingly held” intention on proximate and remote targets (discussed below), William Tiller has developed a “reference frame” in which Consciousness is explicitly incorporated into a model of reality involving D-space (spacetime—involving subluminal velocities [**v** **<** **c**)] and R-space [quantum vacuum—superluminal velocities (**v** **>** **c**)]. Tiller expresses the creation relations among the three as a “general reaction equation” (**Equation 1**).^30^

**Equation 1**. Tiller's general reaction equation.

**Figure:**
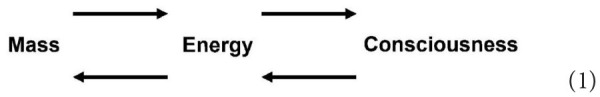


In his assessment of Tiller's work, Murray Gillin writes that “[T]he importance of Tiller's research [is] to open up a type of ontology that sees *consciousness and matter as two-complementary aspects of one reality* [which enables a] fuller understanding of human intention ….” (Gillin, 2021, p. 236–237; italics added). This is apparent in Tiller's model (see Figure 7) where Consciousness is conceptualized outside physical reality as a series of successively “higher” dimensions—Emotion, Mind, and Spirit. Working synergistically, they are the means by which intention exerts its will on Matter.

However, while a significant improvement on previous efforts in this regard, Tiller's approach is hobbled by some unfortunate problems,^31^ of which the most important is his use of Claude Shannon's concept of information—*reduction of uncertainty* (Shannon, 1949). Shannon's informational unit—the BIT—is a binary digital element used to code and communicate logical or symbolic information, *only*: it cannot encode structural information directly from a source object. Thus, Shannon's concept is *not* able to describe *how* figural/structural features of objects/events are communicated in psychoenergetic (or any other) interactions. In the present context, these include the images of objects/events perceived in remote viewing, precognition, intuition, channeling, and so forth, or the image of intention/healing communicated to the target during focused intention (as illustrated in CT's psychic communication with her son). By contrast, as described next, Gabor's unit of information—the *logon*—captures these features because they are encoded in the scatter and diffraction wave patterns as energy encounters objects/events. This, along with Pribram's quantum holographic research on memory and perception in the brain (Pribram, 1991, 2013), among other considerations (see Bradley, 2024c), led RB to revamp Tiller's model of reality in the terms of a quantum holographic approach (Figure 7), which is outlined, momentarily.

In a recent anthology, Marks-Tarlow (2020); Marks-Tarlow et al. (2020) explore the utility of “fractal self-similar resonance” as a potential mechanism in transpersonal psychology for relaying information between macro and microscale levels (see Marks-Tarlow, 2020), including that involving psychic *imagery*—both real and imagined (dreams/nightmares, hallucinations, and so forth). Their approach is based on Mandelbrot's (1977) ground-breaking classic, *The Fractal Geometry of Nature*, which describes a “new” mathematics underlying the repeated “irregularities” and “fragmentation” patterns observed across scale in nature (Mandelbrot, 1977, p. 21). Encompassing both the “fractal face” of nature's patterns—the objects of perception—and the geometry of psychic imagery, we posit that the *spectral signature of a fractal “object”* is encoded in the movement of energy, via a holographic process. In this way, *structural/figural* information on patterns and dynamics are communicated across scale, as related below.

### A quantum holographic approach^32^

As we will see, because quantum holography pertains to the encoding of information across the *entire* frequency spectrum—viz, those of spacetime *and* the quantum domain—it provides a two-way transmission bridge for energetically encoded information communication *between* them (Figure 7). This property of quantum holography is the basis of its wide-ranging application in imaging/scanning technologies (such as, *f* MRI, CT, and CAT scans, plus spectral, sonographic, and acoustical scanning technologies) in such fields as medical science, engineering, earth science, and astrophysics (Schempp, 1992). Quantum holography is also viewed as “‘the basis for the interface between mind and matter” (Mitchell, 2004). We begin with a distinction between Classical and Quantum Holography.

### Classical and quantum holography

**Figure 8:**
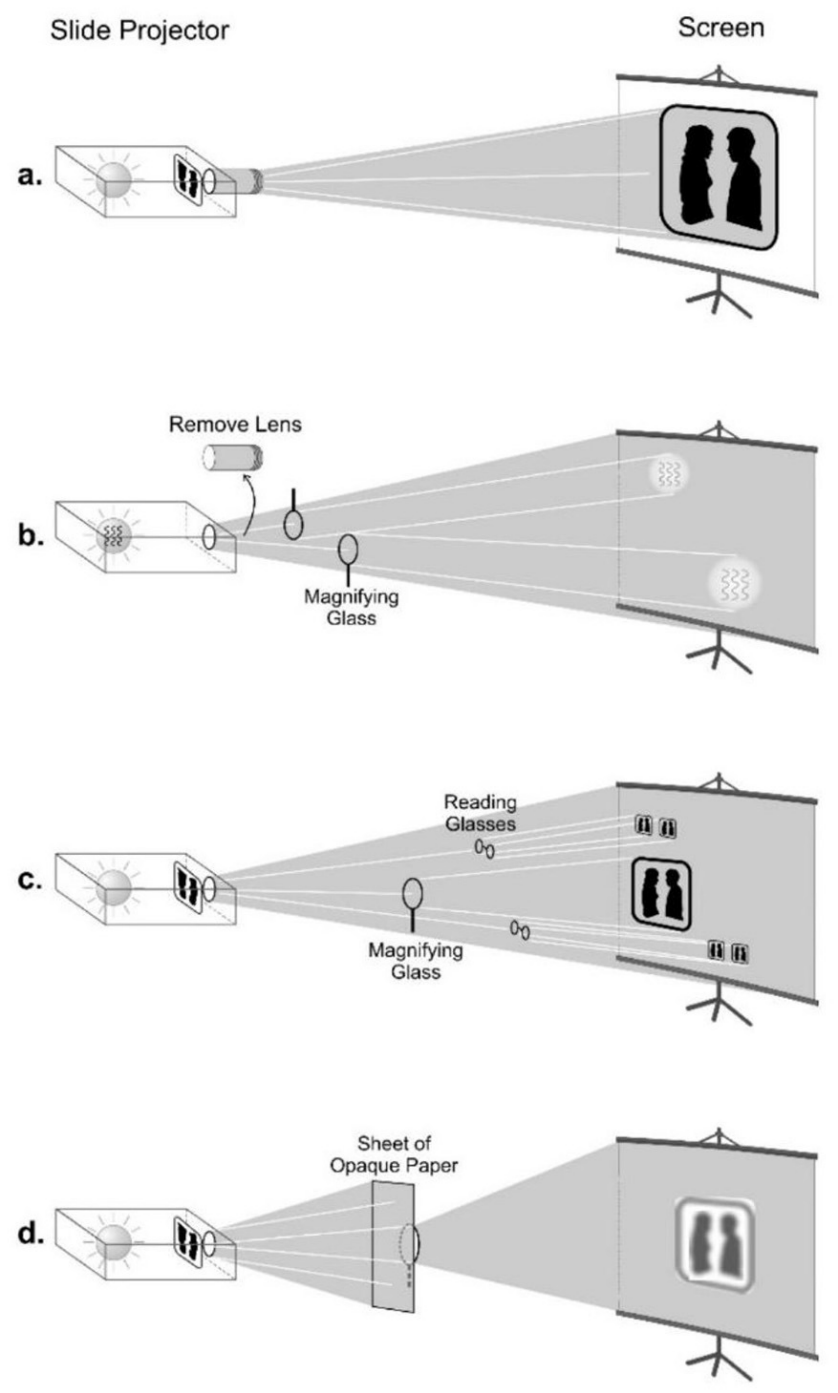
Demonstration of a holographic effect. The normal setup for a slide projector **(A)** is shown first, with the image of a slide projected by the light emitted from the light bulb (and focused by) the lens onto the screen. In **(B)** both the lens and the slide are removed, so that a cone of empty light “white” light is projected onto the screen—“empty,” in that no image of anything is apparent. However, holding a magnifying glass perpendicular and close to the projector, at the right focal length, reveals an image of the light bulb's filaments wherever the magnifying glass is held in the plane of the light cone field projected to the screen. In short, the features of a source object (in this case the light bulb) are spectrally encoded as an image everywhere in the energy emitted from that object. In **(C)** the slide is reinserted, but without the lens, so that the light passing through the slide appears on the screen as “white” light; no features or image of the slide are visible. However, holding a magnifying glass and/or one more pairs of reading glasses (each is a lens), an image of the whole slide can be retrieved can be retrieved for each lens, at the appropriate focal length, from *anywhere* within the cone field of “white” light—demonstrating that an image of the slide is encoded *everywhere* throughout the light's energy field. Finally, covering most (~90–95%) of the magnifying glass lens with a sheet of opaque paper, as shown in **(D)**, the slide's image can still be retrieved from *any* position in the light cone field—demonstrating the holographic property of information redundancy. The image's features are fuzzy, due to the reduced energy density storage capacity, which produces a loss in image resolution. Yet the object, as a whole, is still discernible from the small amount of light energy passing through the tiny portion of the magnifying glass lens not covered by the sheet of paper (adapted from R. T. Bradley and R. J. Nixon, ©, 2014, Center for Advanced Research; reproduced with permission).

Both forms of holography have their origins in the work of Nobel Laureate, Gabor (1946, 1948, 1953). Holographic organization is a field concept of order in which energetically encoded information about the organization of a system—as a *whole*—is enfolded into the field and distributed, nonlocally, by the movement of energy to all parts and locations. The process occurs in spacetime as energy radiates—scatters and diffracts—from interaction with objects, creating an interference pattern that contains a tacit image encoded (in terms of frequency, amplitude, and phase) in the *spectral domain*, an invisible reality apart from spacetime. The nonlocalized distribution of information makes it possible to retrieve a *static* image—a *hologram*—of the system's global organization from the *information* spectrally encoded in any part or location within the field, as illustrated in Figure 8.

The discussion involves two distinct realms—spacetime reality and the spectral domain—which are related, in that Gabor showed you can get from one to the other and back by means of a Fourier transform (FT) function, which means it is *invertible* (Gabor, 1948). Thus, a *Forward* FT (***F(k)***) into the spectral domain; and an *Inverse* FT (***f(x)***) back to spacetime. Although seemingly paradoxical, mutually exclusive domains, spacetime and the spectral domain are *complementary*: viz, while each embodies a different face of reality, they are unified by a specific physical mechanism of translation—the Fourier transform function.

**Figure 9:**
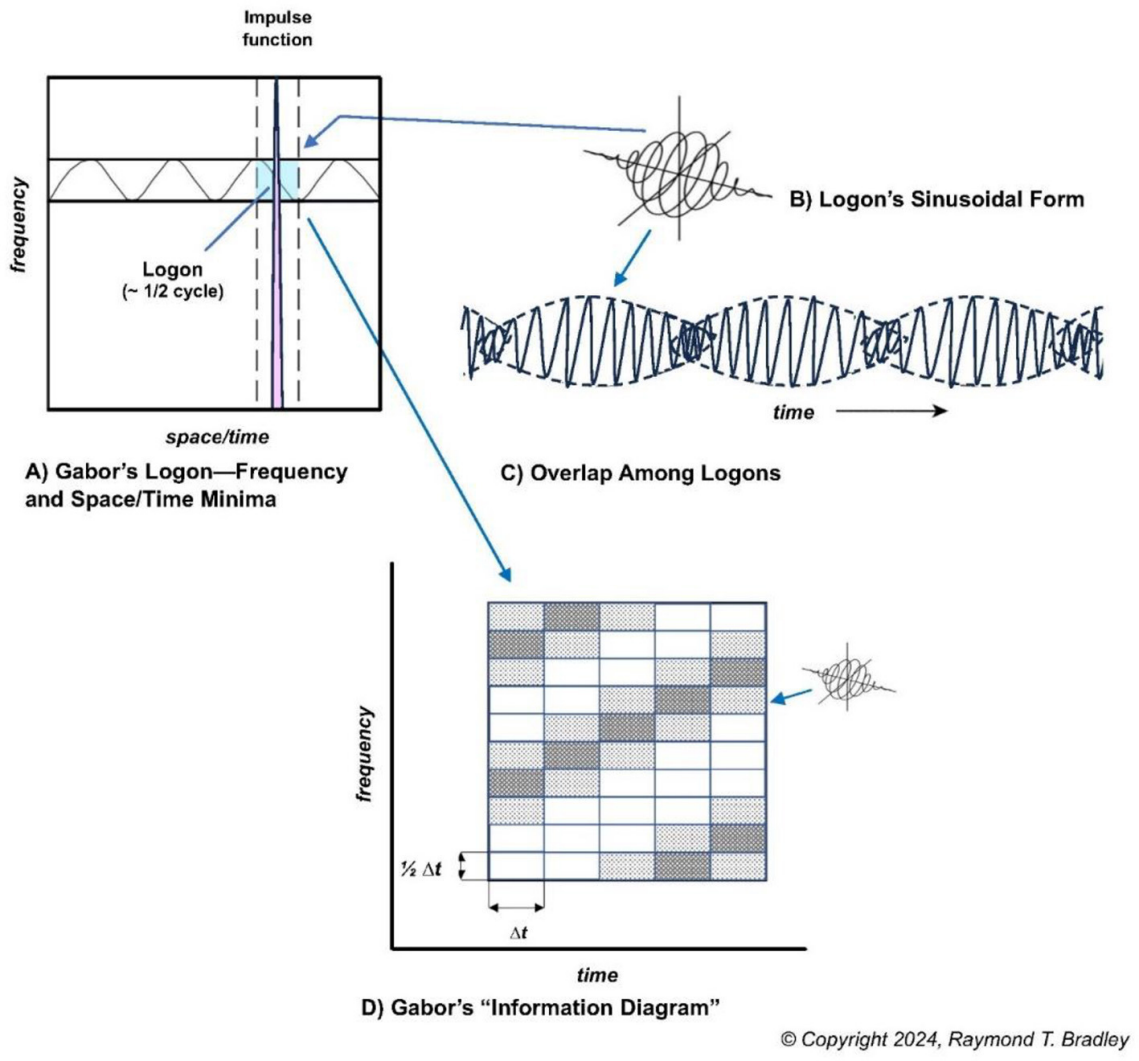
Basic features of Gabor's elementary unit of information—the logon. **(A)** The *minimum* amounts of frequency and space/time (shaded rectangle) required to encode a signal, which Gabor called a *logon*. **(B)** The logon's 3-dimensional sinusoidal form which emerges from the real (cosine) and imagined (sine) components of the complex Gabor waveform (Gaussian modulated). **(C)** The “overlap” of logons wherein the information from a given logon is spectrally enfolded into adjacent logons. **(D)** Gabor's “Information Diagram” per his “expansion” method for grouping ensembles of logons to capture the *entire* signal (see Gabor, 1946, p. 435–437). The pattern of shading, diagonally across the cellular matrix, represents the “peaks” and “troughs” morphology of waves of energy (from Bradley, 2024c, Figure 2, © copyright, Bradley, 2024; reproduced with permission).

These same dimensions, spacetime and frequency, are also the basis of Quantum Holography. Except here, drawing on the mathematics of Heisenberg's principle of uncertainty,^33^ (Gabor 1946) treats the two as orthogonal coordinates in which measurement precision on one ordinate is obtained at the cost of total *uncertainty* on the other. However, when *conjoined*, as conjugate variables, the coordinates create a phase space in which minimum values on both can be mathematically determined (Figure 9A). The phase space is Gabor's elementary unit of information (**φ_*jk*_(*t*)**), these days known as the Gabor elementary function (MacLennan, 1994). Gabor called this area a *logon*, or a *quantum* of information,^34^ and showed that the signal that occupies this minimum area “is the modulation product of a harmonic oscillation [of energy] of *any* frequency with a pulse in the form of a probability function” (Gabor, 1946: 435; italics added). This area defines the *minimum* amounts of energy frequency and space/time required to encode a feature element of a signal for communication, with fidelity.

In Gabor's mathematics, the phase space is Gaussian modulated (“windowed”/constrained) to prevent spectral progression to infinity. Viewed in three dimensions, the logon has a sinusoidal form which emerges from its cosine and sine components (Figure 9B). Communicated as a succession of quantized “snapshots”, logons overlap (Figure 9C). The overlap means there is spectral enfoldment of information among adjoining logons, creating, in Gabor's words, an “*overlap of the future”* (Gabor, 1946, p. 437; italics added)—an important property we draw on below. Finally, in Figure 9, the logon is shown as a “cell” in Gabor's “Information Diagram” (Figure 9D), which is the basis of his “expansion” method for measuring/capturing the “entire signal” for information communication.

### Concepts of consciousness

Since nonlocal intuition is a process by which the body perceives tacit information from a nonlocal source and brings that of emotional significance to attentional awareness for potential action, there is the basic question of how these concepts of holography apply to consciousness. We begin with a distinction between two concepts of consciousness: Universal Consciousness and Conscious Awareness (Bradley, 2024c).

*Universal Consciousness* is Knowledge from Source: immutable Truth, and the Laws and Forces which describe how the universe is organized and evolves. In essence, these are the implicit *constants* of nature and spirit which are omnipresent—often referred to as Source or “the hand of God”—and thus, give *shape* to everything. By contrast, *Conscious Awareness* is the flux of information from internal and external sources, both conscious and unconscious, and includes our bodily sensations, feelings, emotions, perceptions, and cognitions. This ever-changing stream of information reflects the interplay of all factors, forces and elements involved in creating each moment of individual experience. It *in*-forms—gives shape to—our existence and actions within the *dynamics* of an ever-evolving spacetime reality.

While both Universal Consciousness and Conscious Awareness are holographically encoded in the movement of energy, two distinct kinds of holographic processing are involved in neural processing of sensory information (Pribram, 1991, 2013). Because the former encodes a *static*, unchanging record, it is postulated that the processes of Classical Holography are the means of preserving and enfolding the *constants* of Universal Consciousness into everything, throughout the entire universe. On the other hand, because the Gabor elementary function involves the processing of an endless stream of moment-by-moment quantized snapshots—logons–it is postulated that the processes of Quantum Holography record information on the *dynamics* of our actions and interactions in spacetime reality. Pribram clarifies that the Gabor elementary function and the Fourier transform function are *both* involved in neural processing, and that the ‘constraints' on processing in the microstructure substrate are affected by “cognitive influences.”

*The form of receptive fields is not static. … receptive field properties are subject to top-down (cognitive) influences from higher-order brain systems. ….The changes are brought about by altering the width of the inhibitory surround of the receptive fields. Thus both Gabor and Fourier processes are achieved, depending on whether communication and computation or imaging and correlations* [respectively] *are being addressed* (Pribram, 2013, p. 109–110; original italics).

This is a crucial point, for it means that the constraint “window” *can be adjusted*—much like a *lens*—to enhance *signal* processing (via Gabor elementary function) or enhance *image* processing (via Fourier transform function)—a key point we'll come back to.

**Figure 10:**
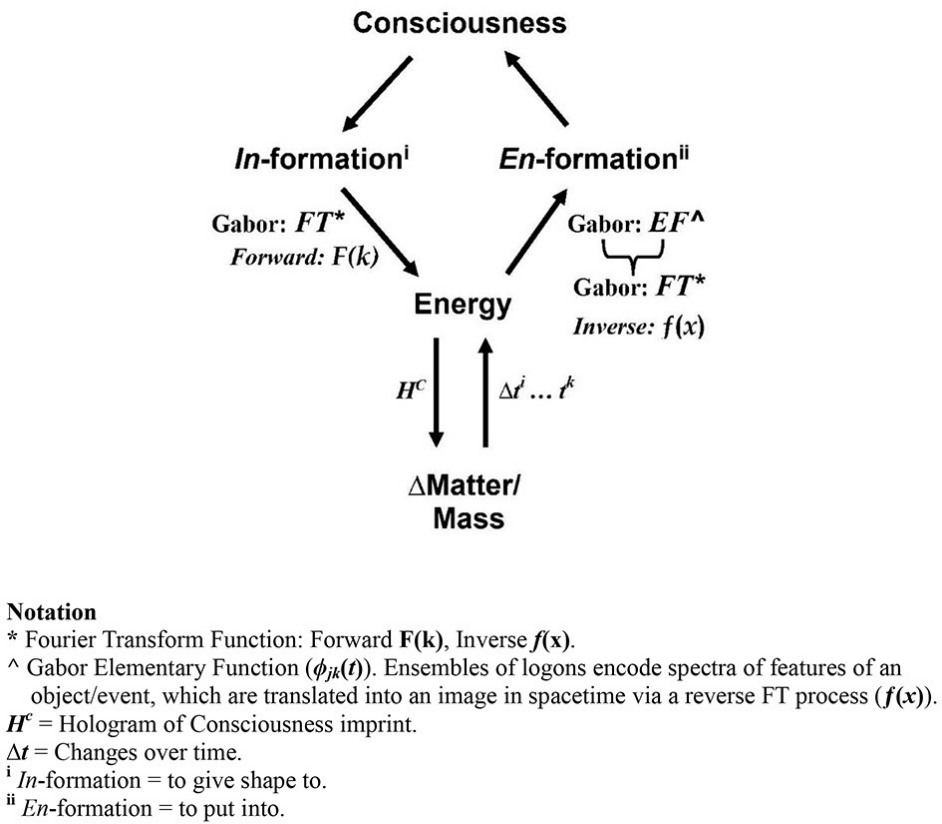
Logic of holographic processes: *In*-formation and *En*-formation. The distinction between two concepts of ‘information' is used to describe the relationship between Consciousness and Matter. The first (*arrows down), In*-formation, means “to give shape to,” per Bohm and Hiley's concept of “active information” (Bohm and Hiley, 1993). Thus, Consciousness—Mind/Spirit—is holographically encoded into the movement of Energy, via a Forward Fourier Transform Function [*F(k)*]—a Classical Hologram, and “*shape”* objects/events in spacetime reality in terms of the frequency signature of the image of order “prescribed”/“desired” in the hologram. The second (*arrows up*), *En*-formation, means “*to put into.”* This is the Quantum Holographic process, via Gabor's Elementary Function (φ_*jk*_(*t*)), which encodes into Energy a continuous record of *all* events and interactions in spacetime as an endless succession of quantized snapshots—*logons*, which are recorded in the ginormous energy store of Universal Consciousness (adapted from Bradley, Figure 6.B, 2024c ©; used with permission).

The distinction between Universal Consciousness and Conscious Awareness points to another fundamental element of reality—namely, *Information*. As depicted in Figure 10, the concept has two meanings. The first, *In*-formation, means “to give shape to.”^35^ Thus Consciousness (including Mind and Spirit) is holographically encoded via a Forward FT. As a hologram, this “*gives shape to”* Matter/Mass in spacetime reality. The hologram encodes the *spectral signature* of the image of order “prescribed” by Consciousness, or willfully induced by an individual's “intention” as an act of agency. The entire process of *psi*-formation is described in detail elsewhere (Bradley, 2024c). The second concept of information, *En*-formation, means “*to put into.”* This is the quantum holographic mechanism, via Gabor's elementary function (Gabor, 1946), for encoding a continuous register of *all* events and interactions in spacetime as an endless succession of quantized snapshots, *logons*. This is recorded in the ginormous energy store of the quantum vacuum—the repository of Universal Consciousness. Applying the two concepts of holography (Figure 10), and drawing on Tiller's work, we have a framework for describing the *creation relations* among the fundamental elements of reality—namely, Consciousness, Information, Energy, and Matter^36^—which we draw on in what follows.

### Love's crucial role

Among Stein's primary conclusions, from her study of women's psychic experience, is the importance of *love*:

Telepathic/empathetic ability runs in generations of women, *linking women together with lines of communication and love*. …. Send your love in any way you know how to express love, and mean it. … Where her love is strong and her thoughts are positive, making the [psychic] link is easier than most women realize. … *distance and space are no barrier between women's minds* (Stein, 1988, p. 195–199; italics added).

In their monumental study, *Margins of Reality*, Jahn and Dunne (1987) arrive at the *same* conclusion to explain the ground-breaking results of their remote viewing (“precognitive remote perception”) and psychokinetic (“telekinesis”) experiments:

Careful application of scientific knowledge and rigor of method, *within a permeating atmosphere of “****love***” in the very general sense … appears particularly pertinent … for the realization of the phenomena themselves …. (Jahn and Dunne, 1987, p. 293; italics and bold font added).

They go on to generalize their conclusions in a basic equation expressing the role of consciousness (Love) as a primary organizing principle in nature (see **Equation 2**):

Whatever may be assigned to this chaos-reducing capability of consciousness, if *L* [**Love**] is its symbol, the process it [**Equation 2**] defines is clearly of the form

(2) $$\begin{array}{l} \text{L}=\;-\Delta s, \end{array}$$

where -Δs denotes the decrease in entropy of the relevant system, the increase in its information, the establishment of its reality (Jahn and Dunne, 1987, p. 339–340; bold font added).

Four decades later, William Tiller and associates published *Some Science Adventures with Real Magic* (Tiller et al., 2005)—a work of enormous importance. The book reports on a 35-year series of experiments documenting the effect of “*lovingly held”* intention imprinted into simple electrical devices (Intention Imprinted Electrical Devices—IIEDs) on various proximate and remote targets,^37^ some as far as 6,000 miles away. They also come to the *same* conclusion on love's vital role in psychic interactions. But they go significantly further, identifying Love as one of the four fundamental elements in the *creation relations* that shape reality (**Equation 3**):

**Figure:**
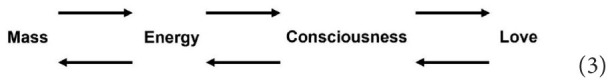


They explain the equation's significance in these terms:

Here, Einstein provided us with the quantitative relationship connecting the first two terms on the left [***E*** **=** ***Mc***^**2**^]…. *The last term on the right [****Love****] is the* ***force of all creation***…*. centering one's consciousness within a framework of* ***unconditional love****, nurturing, caring, etc., can allow the healing process to unfold* from right to left in the above equation *even though we do not know the quantitative connections* (Tiller et al., 2005, p. 230; italics and bold font added).

In short, states of selfless/unconditional love play a pivotal role as the psychoenergetic channel for nonlocal communication and for accessing higher states of consciousness.^38^ As we see next, it is the heart's pattern of rhythmic activity that holds the key, and this can be *intentionally* regulated.

### Heart coherence

**Figure 11:**
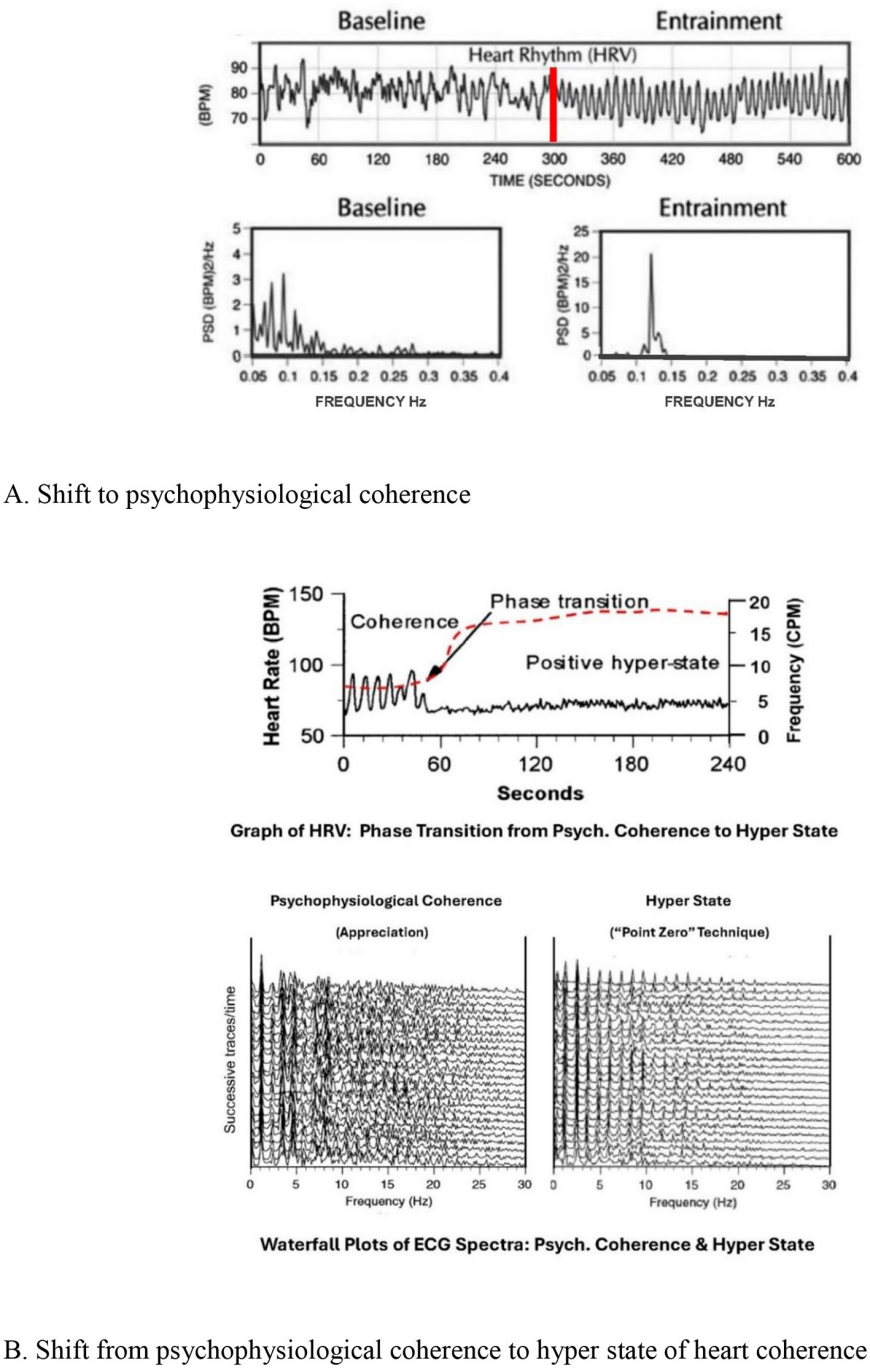
Self-induced shifts to coherence and hyper state. **(A)** The real-time HRV pattern is shown for an individual making an intentional shift from a self-induced state of “frustration” to a selfless loving state (“appreciation”), using an IHM emotional management technique (Freeze-Frame), initiated at ~100 seconds (signified by the red vertical line). Evident is the immediate shift from an erratic HRV pattern (incoherent) associated with their stressful experience of “frustration”, to a smooth, sine-wave-like HRV pattern (coherent) as the individual experiences “appreciation” (adapted from McCraty et al., 2006, © copyright, Institute of HeartMath; used with permission). **(B)** The top graph shows a typical example of the phase transition measured in a subject self-inducing a shift from Psychophysiological Coherence (recorded while holding a loving feeling of “appreciation”) to a positive emotion hyper-state (IHM calls “Emotional Quiescence”), using a “one-pointed” focus meditation technique. Note the abrupt change from the larger-amplitude sine wave-like HRV pattern-signifying Coherence, to the notably higher-frequency (red dotted curve: mean Cycles Per Minute) with a lower amplitude rhythm (black curve: Beats Per Minute), marking onset of the hyper-state. The bottom graphs are “waterfall” plots, displaying the ECG spectra during Psychophysiological Coherence (left) and the Hyper State (right). Whereas, for the former, there is little vertical alignment of the standing waves in the traces during Coherence, a pattern of harmonic progression is seen across the succession of traces recorded in the Hyper State. Each trace in the waterfall plot is the electromagnetic spectrum of the electrocardiogram recording of an individual over a 6-second period. Together, the series of traces cover a continuous period, approximately 2 1/2 minutes (adapted and redrawn from McCraty et al., 2006, © copyright, Institute of HeartMath; used with permission).

A ground-breaking discovery by IHM found that a sustained *loving focus* shifts the heart to a *coherent* pattern of rhythmic activity (Tiller et al., 1996) (Figure 11A). This change—a *phase shift*—entrains the brain and the autonomic system into a state of *system-wide Psychophysiological Coherence*—also known as Heart Coherence (McCraty et al., 2006). As Tiller explains:

When focusing on the heart with *loving intent*, the human EKG becomes *harmonic* at the baroreflex frequency [0.12 hertz], where the heart entrains the brain and simultaneously all the other major electrophysiological systems of the body (Tiller et al., 2005, p. 139; italics added).

This is of direct relevance to mother-offspring intuition, in that above it was posited that heart coherence *mediates* creation of the bond of multiscale entanglement. Supporting evidence is measurement of mother-fetus cardiac synchronization in the womb (Ivanov et al., 2009), and—postnatally, for infants/toddlers—of heart synchronization to the mother's heart pattern when she holds states of selfless love, such as happiness and joy (Schore, 1994; Feldman et al., 2011).^39^

#### Hyper states of emotion

Subsequent investigation by IHM researchers found that *extreme* emotions were signified by an abrupt HRV phase transition to a *hyper state* “discontinuous” from normal psychophysiological states of daily life (McCraty et al., 2006). Thus, from the normal (baseline) state along the positive emotion axis, two distinct phase transitions could be self-induced and were associated with the experience of successively “higher” states of consciousness (Bradley, 2024a). Electrophysiologically, the shift to a Hyper State is signified by a phase transition in heart activity to a higher rhythmic frequency (Figure 11B, top graph, red dotted curve) with low amplitude waveforms. Notably, when measured in terms of frequency (hertz) in the ECG spectra, the Hyper State displays a *harmonic series* (“waterfall” plot, bottom right graph), whereas Psychophysiological Coherence shows little harmonic alignment over the traces (bottom left graph).

To self-induce the phase transition to the Hyper State, a “one-pointed” focus meditation technique is used in which the practitioner adopts a sustained state of selfless/unconditional love (McCraty et al., 2006). In terms of Pribram's (2013) “cognitive influences” on processing in the neural microstructure, noted above (page 17), this widens the inhibitory neural surround toward Fourier processing, thereby facilitating access beyond spacetime (Gabor processing) to higher and hyper dimensions of reality—viz, higher states of consciousness, including Universal Consciousness itself (see Bradley, 2024c). In short, these phase shifts can be self-induced with disciplined practice—as long known by ancient spiritual traditions.

### Passionate attention

**Figure 12:**
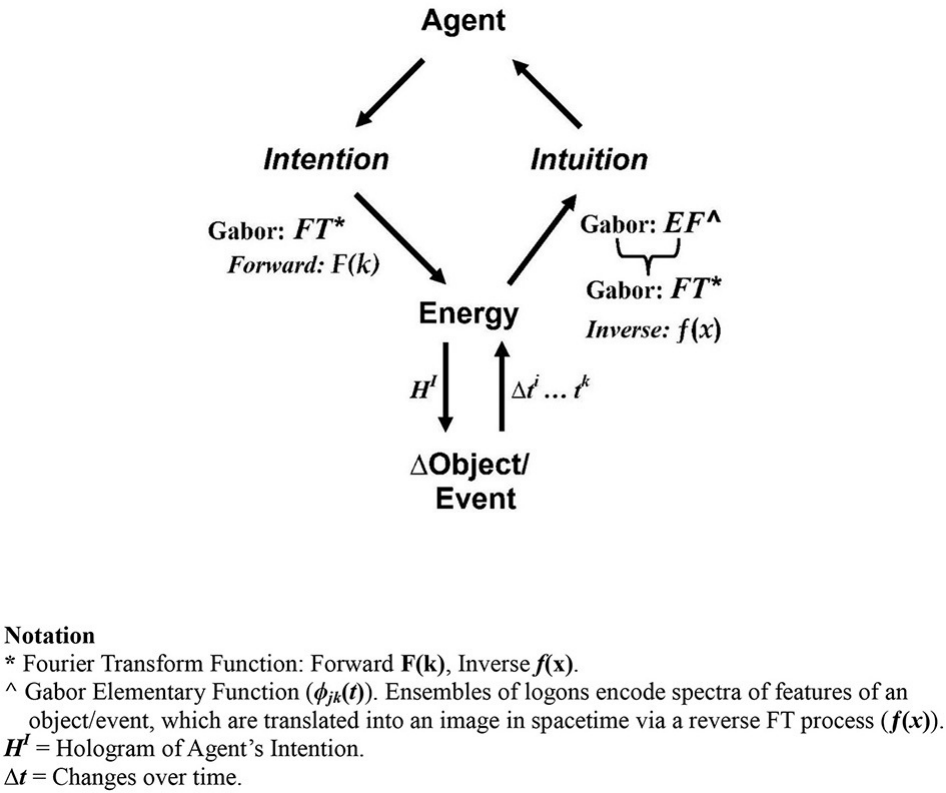
Logic of holographic processes enabling agency. The figure shows the translation of *In*-formation and *En*-formation into the holographic communication processes for Intention and Intuition, respectively. *Left*: the flow logic (**Agent**→**Intention**→**Energy** → Δ**Object**/**Event**) describes the process by which the Agent's Intention is encoded into Energy as a Classical Hologram, via a *forward* FT (***F(k)***), to induce change in the Object/Event). *Right*: the flow logic (Δ**Object**/**Event**→**Energy**→**Intuition**→**Agent**) describes the precognitive processes by which information about change in the Object/Event is quantum holographically encoded into Energy, via the Gabor elementary function (φ_*jk*_**(*t*)**), transmitted back to the Agent/percipient, and decoded-via an *inverse* FT (***f(x)***)-into Intuition in terms of spacetime imagery (from Bradley, 2024c, Figure 10, © copyright R.T. Bradley; used with permission).

Emotional attachment to an object of interest activates the arousal of biological energy required for agency (Figure 12). Thus, tuning into and maintaining resonance with the energetic frequency of the object/event, passionate attentional focus creates a two-way psychoenergetic communication channel between the percipient/agent and the object (Bradley, 2010). Both outgoing and incoming wavefields contain information about their respective sources holographically encoded in the movement of energy. Hence

The incoming wave front carrying information may be labeled as “perception” [or intuition] from the point of view of the percipient, and the return path required by the resonant relationship may be labeled “attention” [or intention] (Mitchell, 2000, p. 302).

Marcer (1995) has shown, mathematically, that a relationship of “*phase-conjugate-adaptive-resonance*” (PCAR) *must* exist between the two wave fields for “accurate” perception of an object in spacetime reality. Thus, PCAR is a process in which the incoming and outgoing wave fields are phase-conjoined by the percipient's act of attention, in that s/he tunes into and maintains an adaptive “vibratory resonance” with the object. In this way, maintenance of PCAR ensures integrity of communication.

Beyond its direct role in agency, passionate attention is determinative of the nonlocal bond of psychic connection between a mother and her offspring. It is the mother's “passion” for her prospective baby—her focus of a (mostly) persistent state of unconditional love on the fetus throughout her pregnancy—that creates the requisite conditions for multiscale entanglement between the pair. Namely, heart coherence.

#### The intuitive heart

The body's life processes generate a multiplicity of wave fields of energy that radiate outwards in all directions and interpenetrate the incoming wavefields of energy from all objects/events—including the universe itself. Both the outgoing and incoming wavefields contain holographic information from the source encoded in the movement of energy. As the most powerful generator of rhythmic activity in the body, the heart's wavefields have enormous reach. When in a coherent state, like a laser, this is vast. Studies show that nonlocal perception is related to the percipient's degree of emotional arousal generated by an object/event (Bancel and Nelson, 2008; Nelson, 2008; Bradley, 2020). Thus, *it is the energetically more powerful, emotionally arousing component of attention*, rather than the mental (purely cognitive) element, *that is propelling the outgoing wave field of bio-emotional energy*. As shown in the IHM study, the heart plays a significant role in the body's sensing and processing of precognitive information, in that the heart receives information about remote/future events *before* the brain (McCraty et al., 2004a,b). Once received by the brain, the prestimulus information is decoded by a reverse Fourier transform process (***f(x)***), as described by (Pribram 1991), and translated into mental imagery, feelings, and other sensations in our Conscious Awareness,^40^ which registers the experience as an *intuition*.

### Quantum holographic description of nonlocal intuition

Finally, the logic of processes involved in nonlocal intuition can be schematically summarized (**Equation 4**).^41^

**Equation 4.**

**A. With Multiscale Entanglement**


$$\begin{array}{l} \left(\left(\text{A}↔\text{O})({\text{L}}^{ti...tk}\right)\right)\to \{\text{ME}\psi^{\left(\text{A}↔\text{O}\right)}(\text{QH}\phi^{\left(\text{SpT}↔\text{QD}:v=i\right)}{\text{+HC}}^{\text{HS}}\\ \text{=}>[{\text{PCAR}}^{\left(\text{A}↔\text{O}\right)}\to {\text{O}}^{\Delta t\text{/}fp}\\ \text{=}>\text{QH}\phi {\text{O}}^{\Delta t\text{/}fp}\to \text{A})]\}\\ \text{=}>\text{A}(\text{f}(\text{x}))\;\text{Intuition} \end{array}$$

Where ***v* =** ***i***, means velocity (***v***) of the signal is instantaneous (***i***)—see Table 1 for notation.

**Table 1:** **Equation 4** notation—Terms and definitions.

**Terms**	**Definition**
**Notation**	
A	Agent.
O	Object/percipient.
L*^*ti*…*tk*^*	Sustained unconditional Love, over time.
{MEψ…}	Field of Multiscale Entanglement in which communication is instantaneous (***t** **=** **0***), irrespective of distance (***d** **=** **∞***).
(QHφ^(SpT↔*QD*)^…)	Quantum holographic logon ‘bridge' between SpaceTime and the Quantum Domain.
= > HC^HS^	Phase transition to Heart Coherence Hyper State.
= > [PCAR^(A↔*O*)^…]	Phase transition to and maintenance of Phase Conjugate Adaptive Resonance relation between Object/Percipient.
O^Δ*t*/*fp*^	Change over time and/or change in future potentials of Object.
= > QHφO → A	Encoding of Object changes into a Quantum Holographic signal with continuous communication to Agent.
= > A(f(x)	Transformation of hologram, via an *inverse* FT (**f(x)**), of precognitive spectra into spacetime imagery enabling Agent's perception as Intuition.

**B. Without Multiscale Entanglement**


$$\begin{array}{l} ((\text{A}↔\text{O})({\text{L}}^{ti....tk}))\text{=}>{\text{HC}}^{\text{HS}}\\ \text{=}>[{\text{PCAR}}^{\left(\text{A}↔\text{O}\right)}\\ \left(\text{QH}\phi^{\left(\text{SpT}↔\text{QD}:\text{v}>\;\text{c}\right)}\to {\text{O}}^{\Delta t\text{/}fp}\text{=}>\text{QH}\phi {\text{O}}^{\Delta t\text{/}fp}\to \text{A})\right]\\ \text{=}>\text{A}(\text{f}(\text{x}))\text{Intuition} \end{array}$$

Where **v** **>** **c**, means velocity (***v***) of the signal is greater than the speed of light (***c***)—see Table 1 for notation.

### Practice implications

Based on the above elaborations and drawing from Bradley's work elsewhere (Bradley, 2020, 2024b,c), we suggest five Principles of Nonlocal Interaction which may have utility in guiding and honing psychic proficiencies—both in women and men—and serve to inform research as hypotheses:

- **Principle 1:** Our bio-emotional emissions interconnect us to everything around us, proximate or distant, which means that we influence and are influenced by all systems-including the Universe itself-whether, consciously, we intend to or not.
- **Principle 2**: By holding a loving state in our daily lives, we are energetically attuned to everything around us and can receive intuitive insights and creative inspirations which can guide our decisions and actions.
- **Principle 3**: Holding deeper states of unconditional Love, by sustained meditation, prayer, or deep selfless introspection, we can access spiritual Truth, the Laws of Nature, and the Source of everything.
- **Principle 4:** Holding sustained focused intentions, during a state of unconditional Love, we can heal ourselves and others and induce change in material and living systems, and in doing so even change the world.
- **Principle 5:** Nonlocal effects (intuition and intention) are greatly amplified by collective energetic resonance generated by a commonly held focus in mass social aggregations and in groups with high socioemotional coherence.

### Summary

To explain the nonlocal information communication processes involved in mother - offspring intuition, we drew on RB's quantum holographic (QH) approach. Based on Gabor's concept of an elementary unit of energetically encoded information—the *logon*, quantum holography spectrally encodes structural/figural features of objects/events in an ongoing succession of quantized “snapshots.” Because Gabor's concept of information applies to *any* frequency, logons are communicated across the “Great Divide” in physics—viz, the “gap” between spacetime and the quantum domain. Since it encodes structural/figural features of objects/events, QH captures emotional valence and images of the source object/event in the intuition communication signal. This includes the *spectral signature* of *fractals* encoding Mandelbrot's geometric “face” of Nature's “self-similar” orders across scale, including that of psychic imagery.

As shown in the IHM experiment, the heart receives the prestimulus information a second or so before the brain, and upon reception by the brain this information is translated, via an inverse Fourier transform function (***f(x)***), into emotional information and imagery for conscious perception as an intuition. Finally, the essential role of selfless/unconditional love and the hyper state of heart coherence in creation of a psychoenergetic transmission channel between percipient/s and the nonlocal object/event, was documented—with the caveat that PCAR must be maintained for communication integrity to ensure efficacy of nonlocal communication.

## Conclusion

Throughout this work, we have provided a summary of the major conclusions and so will not repeat these here. Instead, we want to highlight the key difference between the concepts of quantum entanglement and multiscale entanglement and elaborate how the latter is constructed to provide a more adequate understanding of human non-local communication, such as mother offspring intuition, and other psychic phenomena. But first, there is the question of men.

Given our focus on women, we have said little about non-local intuition in men, aside from the comparison results in the few studies by gender. While it is expected that some of the differences observed in prestimulus response, psychophysiological system involved (such as the “gut” vs. the “heart”), socioemotional sensitivity, and so forth, may be more pronounced among some men, it is likely that changes in gender roles are exerting a modifying influence. As men in recent generations play a more active, even primary role in parenting, there is evidence that the postnatal socioemotional attunement, documented above between a mother and child, can be induced by a loving father/parent, more generally (Feldman, 2025; Feldman et al., 2010). Insofar as the “good enough” father/parent maintains a mostly persistent bond of selfless love with the child, the process of multiscale entanglement will likely create a channel of psychic connection between them. Equally, as women have moved to embrace roles involving greater physicality and increased risk of injury/danger (such as in sports, the military, and so forth), concomitant adaptions in psychophysiological basis, intuitive capability, and socioemotional sensitivity are anticipated.

Turning to the concepts of quantum and multiscale entanglement, it is their difference in scope-the phenomenon each encompasses-that is key in relation to understanding human psychic proficiency. As we noted, “Quantum physics views all matter, entities, force fields, and energy at this level of reality as *inert*....” (p. 11). This means that quantum entanglement in itself cannot explain nonlocal biopsychosocial or psychic communication.^42^ To do that, the entanglement concept must include the very elements that physics excludes: namely, *life, sentience, agency, and consciousness*!

We then clarified that “By comparison, multiscale entanglement is a broader concept encompassing the microscale of quantum phenomena, the macroscale domain of biophysiological reality, *and* the higher dimensional spaces of psychosocial and psychic reality” (p. 11). In other words, we aimed to construct a larger concept of entanglement, encompassing the levels of reality and processes involved, that not only captured quantum reality and the physical and biopsychosocial elements that define the world of life, but also the psychic and spiritual dimensions where consciousness resides as a domain apart. Building upon Tiller's work toward this goal, RB incorporated classical and quantum holographic concepts of image and information processing, respectively, into a wholistic framework (Figure 7, p. 15) integrating all these aspects of reality. This provides the conceptual means to adequately address human nonlocal communication. Using this approach the enigma of mother-offspring intuition can be understood. Thus, by showing *how* a psychic bond of instant intuitive communication is created and operates between a mother and her offspring, multiscale entanglement provides a rational (scientific) description of the micro to macroscale processes involved.

In addition to its utility on this question, we also found that multiscale entanglement appears to have relevance to the transmission of transgenerational trauma. CT had delved into several studies on this puzzling phenomenon, following an intuitive hunch of a connection to multiscale entanglement. In our deliberations about this, an unanticipated insight crystallized in the form of a novel hypothesis: namely, that multiscale entanglement provided a potential psychic mechanism for explaining how emotional upset and mental imagery associated with transgenerational trauma could be transmitted to progeny of later generations—not only across many prior generations of those “long dead,” but also between adjacent generations.^43^ In practice, the therapist might consider transgenerational trauma when evaluating those “hard to heal” cases.^44^ Making this connection, when appropriate, can be transformational for a client. When ill, either physically or emotionally, we search for answers and understanding. In the case of transgenerational trauma, once known, the client is empowered to choose a new reality, thereby opening a path to true and lasting healing.

Beyond these applications, it is likely that multiscale entanglement is the basis of the nonlocal channel of psychic connection among species of social animals (including plant communities) and likely mediates the psychic bond between humans and their pets and with wild animals (Sheldrake, 1999; Bradley, 2012). Undergirding *all* of this, is the crucial role of selfless/unconditional love in establishing and facilitating such nonlocal bonds.

A final word. The science of intuition, we have laid out here, is a road map of practice principles for how to begin this incredible journey—one of discovery and growth, for both women and men. These are embodied in one simple practice, which is a lifetime endeavor, that we try to follow in all our actions: *Always do the most loving thing*.^45^

But there is an important group of adepts in a category, all their own—psychic and spiritual “masters”—for whom no gender differentiation is expected, either on a psychophysiological basis, degree of psychic/spiritual proficiency, and so forth. As long understood by ancient spiritual traditions, this is because there is only *one* path to the psychoenergetic channel that opens these domains of reality and Truth: namely, a sustained state of Unconditional Love:

The virtue from the hand or breath may heal a thousand more; but *love is queen*. Thought, reinforced by love, is God's great sovereign balm (Levi, 1907, p. 42; italics added).

## Data Availability

The data analyzed in this study is subject to the following licenses/restrictions: The data were gathered and held by the Institute of HeartMath. Requests to access these datasets should be directed to Dr. Rollin McCraty (rollin@heartmath.org), Director, Research Center, Institute of HeartMath.

## Footnotes

^1^As depicted in Figure 1, “calm” stimuli (represented by the “rabbit” image) included pictures of flowers, pets, peaceful scenes/landscapes, and so forth. “Emotional” stimuli (signified by the “snake”) included pictures of menacing wild animals, threatening scenes, etc.

^2^RPA is performed to control for autocorrelations inherent in physiological signals. It provides a *conservative* measure of statistical significance (*z* statistic) of differences between emotional and calm prestimulus response.

^3^See the reviews of research in Sinclair (2020); also, Holm and Severinsson (2016); Bao et al. (2022).

^4^Bao et al. (2022) note that whereas the P3b component “refers to the satisfaction of expectations”, the “N2 component refers to conflict detection with a frontal-central distribution … and is induced by incongruent stimuli.”

^5^See McCraty et al. (2006, p. 40–43).

^6^We note, that while the major finding of the heart's involvement is widely reported in the literature, less attention has been paid to the results by gender.

^7^“When signal averaging techniques are used to trace the flow and processing of afferent neurological signals from the heart through the different regions of the brain, the resulting waveforms are called *heartbeat-evoked potentials*. The peak of the ECG R-wave is used as the timing source for the signal averaging process” (McCraty et al., 2004b, p. 329).

^8^This is aptly captured in Diane Stein's title for her book, All Women Are Psychics (Stein, 1988).

^9^See the review of studies in Radin (2006).

^10^Schore (2003), see Chaps. 7 and 8.

^11^CT: To ensure anonymity, pseudonyms have been used for my son and my two clients (below). Also, any identifying information—such as, geographic location and life circumstances—have been removed/changed to protect confidentiality.

^12^CT: My eldest son has given me permission to relate some of our story, as an illustration of the reality of a strong mother-child psychic bond.

^13^See Nolte (2022) for a nontechnical chronology.

^14^The experiment involved two *atoms* each separated in laboratories 400 meters apart using the “entanglement swapping” protocol (see https://xqp.physik.uni-muenchen.de/research/atom_photon/entangling_atoms/index.html).

^15^'Bucky's ball' comprising some 60–70 carbon atoms, was used by Zeilinger's team in their experiment with a ‘double-slit' setup.

^16^See Freedman and Clauser (1972); Aspect et al. (1981); Zeilinger (2017).

^17^Emoto (2003) has investigated and developed “hado [subtle energy] medicine” which is based on the same principle of micro-macroscale resonance in emergent systems as the concept of multiscale entanglement (see p. 21–38).

^18^Space constraints mean we can only deal with the first of these; the other two will be taken up in a subsequent work.

^19^See the review of evidence in Hameroff and Penrose (2013).

^20^This is despite Roger Penrose's efforts (and others) to find a way of introducing ‘consciousness' –sentience and will—into the reference frame of physics (see, Penrose, 1994, 2004; Hameroff and Penrose, 2013).

^21^Or other selfless positive emotions, such as happiness, joy, and gratitude.

^22^Heart-brain synchronization has been documented in studies by IHM (see McCraty et al., 2006). Here we posit it as a mechanism regulating embryonic neural development.

^23^We acknowledge Reviewer 3 for this suggestion.

^24^There is evidence that bond formation may be disrupted chemically by drugs administered to the mother during childbirth (see Gambotto-Burke, 2022).

^25^Maté (1999), describes how the psychological impact of a mother's transfer of anxiety/depression/trauma to a fetus/child produces ADD or ADHD in childhood or adult life.

^26^I suggested Ella talk to her mother about what we discussed, thinking she would likely be relieved that there was an explanation for her daughter's anxiety—including hope for her daughter's mastery over this.

^27^Because space constraints confine us to be brief, the matters in this section will be addressed in a subsequent work.

^28^According to the CDC, “Epigenetics refers to how your behavior and environment can cause changes that affect the way your genes work” (https://www.cdc.gov). Unlike genetic changes or mutations, epigenetic changes are reversible, and although they do not change the sequence of DNA bases, epigenetic changes can change *how* the body *reads* a DNA sequence. Symptoms of transgenerational trauma can be extreme and varied, often debilitating. They may include (but not limited to) *shame, anxiety, guilt, vulnerability/sensitivity, helplessness, hopelessness, low self-esteem, depression*, suicidality, substance abuse, dissociation, *hypervigilance, intrusive thoughts, difficulty with relationships and attachment to others*, difficulty regulating aggression and *extreme reactivity to stress*. Italics are used to show which symptoms appear in CT's case study on “Joseph,” below.

^29^Further documentation of psychic transmission of trauma from a mother to a child, is provided in a George Noory interview (Beyond Belief, Season 27, Episode 7–Telepathy: The Unspoken Gift of Autism), in which William McKenna recounts the case of a New Zealand woman's traumatic experience as a five-year-old child on a sheep farm tasked with cleaning up the entrails and feces of slaughtered lambs. Years later, as an adult, the woman's trauma was psychically sensed and expressed, by her young autistic daughter, as ‘faecal smearing' on the walls of their house. This behavior pattern instantly ceased upon the mother's acknowledgment and ‘clearing' of her trauma source in a treatment session using McKenna's ‘Cognomovement' technique (https://www.giai.com/video/telepathy-the-invisible-gift-of-autism; accessed June 4, 2025).

^30^Tiller et al. (2005), Equation 6.1.

^31^Discussed in depth elsewhere (Bradley, 2024b,c). Also, see (Gabor 1953).

^32^Building on RB's earlier theory (Bradley, 2007, 2010), this section draws heavily on his recent work (see Bradley, 2024a,b,c,e).

^33^Which is defined as Δ*pΔx*>*h*, where Δ*p* is the uncertainty of momentum, and Δ*x* is the uncertainty of location, and *h* is Planck's constant. For a discussion of Heisenberg's ‘uncertainty principle' in English see, W. Heisenberg, Remarks on the origin of the relations of uncertainty, in: W. Price and S. Chissick (eds.), *The Uncertainty Principle and Foundations of Quantum Mechanics. A Fifty Years' Survey*, J. Wiley & Sons, London, 1977, p. 3–6. For the original publication in German see, W. Heisenberg, Uber den anschaulichen Inhalt der quantentheoretischen Ki ¨ nematik und Mechanik, Z. *Phys*. 43 (1927) 172–198.

^34^Hence, Pribram's (1991, Lecture 2) characterization of this form of holography as “quantum holography.”

^35^This is Bohm and Hiley's(1993) concept of “active information.”

^36^In acknowledging that this is Willaim Tiller's framework (Tiller et al., 2005), we note that the translation into holographic concepts is RB's (see Bradley, 2024b,c).

^37^Such as room temperature of a controlled space, the pH behavior of water, and fruit fly larvae maturation.

^38^Bradley (2024b, see p. 20–25, especially Figures 7, 8).

^39^It should be noted that postnatal “synchrony” has been documented between fathers and infants (Feldman et al., 2010).

^40^It is in the neural processes of “thinking” (comprehension and meaning) that Shannon's concept of information—reduction of uncertainty—comes in: “*Shannon's formulation allows us to define precisely Decartes's “cogito,” thought, in terms of communication theory*... [it] provides us with at least one scientific measure of the thought process” (Pribram, 2013: 494–495; italics added).

^41^For the details, see Bradley (2024c, p. 20–29).

^42^This is recognized by Tiller who proffers the notion of “information entanglement” to redress this limitation (see Tiller et al., 2005, p. 196–210). Although lacking explicit definition, RB notes (elsewhere) that Tiller's concept “appears strongly imbued with holographic properties” (see Bradley, 2024c, p. 16).

^43^As documented in the studies cited above (Costa et al., 2018; Gapp et al., 2020; Yehuda, 2022) and illustrated in CT's cases (Ella and Joseph) and the example described in Footnote 29 of the New Zealand mother's trauma transmission to her child.

^44^CT applied this concept to a puzzling case of a woman who exhibited extreme paranoia and fear about her health, even though overall, she was healthy. It was discovered that her adult son has been dealing with serious life-threatening health problems. CT observed that when he was feeling healthy and positive, the client would also function better. Yet when the son was in crisis, the client would develop fears of her own physical health, often to the point of emergency room visits and tests. The possibility of there being some transgenerational trauma “entanglement” occurring in their relationship seems high and is currently being explored.

^45^This is Joseph Fletcher's maxim. The scientific basis for this universal principle of spiritual practice and its implications as a guiding principle for individual and collective life are described at length in *The Lens of Love* (Bradley, 2024d).
